# Therapeutic Interventions for Vascular Parkinsonism: A Systematic Review and Meta-analysis

**DOI:** 10.3389/fneur.2017.00481

**Published:** 2017-09-22

**Authors:** Adán Miguel-Puga, Gabriel Villafuerte, José Salas-Pacheco, Oscar Arias-Carrión

**Affiliations:** ^1^Unidad de Trastornos del Movimiento y Sueño (TMS), Hospital General Dr. Manuel Gea González, México City, México; ^2^Plan de Estudios Combinados en Medicina (PECEM), Facultad de Medicina, Universidad Nacional Autónoma de México, México City, México; ^3^Instituto de Investigación Científica, Universidad Juárez del Estado de Durango, Durango, México; ^4^Centro de Innovación Médica Aplicada (CIMA), Hospital General Dr. Manuel Gea González, México City, México

**Keywords:** vascular parkinsonism, therapy, treatment, systematic review, meta-analysis

## Abstract

**Background:**

Vascular parkinsonism (VP) is defined as the presence of parkinsonian syndrome, evidence of cerebrovascular disease, and an established relationship between the two disorders. However, the diagnosis of VP is problematic, particularly for the clinician confronted with moving from diagnosis to treatment. Given the different criteria used in the diagnosis of VP, the effectiveness of available therapeutic interventions for this disease are currently unknown.

**Methods:**

To assess the clinical response of all published therapeutic interventions for VP that have been reported in the literature, we conducted a systematic review looking for VP subjects treated with any therapeutic intervention. To clarify the prevalence of responsiveness to levodopa among VP subjects, we conducted a meta-analysis of 17 observational studies retrieved with the search criteria of our review. Also, four studies were included in a second analysis to explore if nigrostriatal lesion affected the prevalence of levodopa response in VP subjects. Relevant articles were identified from MEDLINE, Scopus, and Web of Science published until June 2017.

**Results:**

436 non-duplicate citations were identified for screening, 107 articles were assessed for eligibility, and only 23 observational studies were included in this review. No randomized clinical trials were found. Four different therapies were found in the literature; among them, levodopa was the only one repetitively reported. The calculated event rate of levodopa response in VP subjects was of 0.304 [95% confidence interval (CI) of 0.230–0.388]. The overall odds ratio for good response to levodopa in VP with lesion in the nigrostriatal pathway vs. no lesion in the nigrostriatal pathway was 15.15 (95% CI: 5.2–44.17).

**Conclusion:**

Despite the lack of randomized controlled trials, results of this systematic review and meta-analysis show that VP subjects, as operationally defined here, have a low response rate to levodopa; nigrostriatal lesion could be used as a proxy predictor of levodopa response in VP subjects. Other therapies seem to be co-adjuvant. Randomized controlled trials with a clear definition of VP are necessary to be able to assign positive or negative predictive values to available treatments and to recommend any of the therapeutic interventions for these subjects.

## Introduction

Vascular parkinsonism (VP) is defined as the presence of parkinsonian syndrome, unequivocal evidence of cerebrovascular disease, and an established relationship between the two disorders (1, 2). The predominantly reported clinical manifestation of VP is lower-body parkinsonism (mainly impaired gait with unstable posture, poor response to levodopa, difficulty maintaining balance and frequently, exhibition of freezing) (3). Imaging studies have been used to corroborate the diagnosis of VP, but given the prior probability of vascular disorders in the older population, these studies established correlation but not causation (4). The criteria of Zijlmans et al. (1) (Table 1) moves toward establishing causation by imposing time constraints between the diagnosis of parkinsonism and imaging, although it is difficult to precisely ascertain the onset of parkinsonism. Ever since the concept of VP was first introduced by Critchley in 1929 (5), its existence has been subject to debate due to the lack of consensus regarding its diagnostic criteria. The broad spectrum of reported cases has been variably referred to in the literature as arteriosclerotic parkinsonism, arteriosclerotic pseudo-parkinsonism, pseudo-VP, vascular pseudo-parkinsonism, and lower-body parkinsonism, even when the physiopathology differs from that of VP (1, 2, 6).

**Table 1:** Zijlmans’ vascular parkinsonism criteria.

Zijlmans’ diagnostic criteria^1^
**Step 1. Parkinsonian syndrome**
Bradykinesia
At least 1 of the following:
Rest tremor Muscular rigidity Postural instability not caused by primary visual, vestibular, cerebellar, or proprioceptive dysfunction
**Step 2. Cerebrovascular disease** Evidence of relevant cerebrovascular disease by brain imaging: CT or MRI.
*AND/OR*
Presence of focal signs or symptoms that are consistent with stroke.
**Step 3. An established relationship between the parkinsonism and the cerebrovascular disease** Acute VP: an acute or delayed progressive onset with infarcts in or near areas that can increase the basal ganglia motor output (GPe or substantia nigra pars compacta) or decrease the thalamocortical drive directly (VL of the thalamus, large frontal lobe infarct). The parkinsonism at onset consists of a contralateral bradykinetic rigid syndrome or shuffling gait, within 1 year after a stroke
OR
Insidious VP: an insidious onset of parkinsonism with extensive subcortical white matter lesions, bilateral symptoms at onset, and the presence of early shuffling gait or early cognitive dysfunction
**Step 4 Exclusion criteria for VP** History of repeated head injury Definite encephalitis Neuroleptic treatment at onset of symptoms Presence of cerebral tumor or communicating hydrocephalus on CT or MRI scan Other alternative explanation for parkinsonism

*CT, computed tomography; GPe, globus palidus extern; MRI, magnetic resonance imaging; VP, vascular parkinsonism; VL, ventrolateral*.

Although the definition of this clinical syndrome is controversial, according to literature, it accounts for approximately 4.4–12% of all patients with parkinsonism (7, 8). The incidence of VP is expected to rise due to an increasingly aging population and the heavier burden of vascular risk factors this entails (9). However, except for controlling vascular risk factors, there is currently no first-line treatment for patients with VP (3).

Levodopa is the most effective treatment for Parkinson’s disease (PD), and in spite of the clinical similarities between VP and PD, VP has been widely characterized as a parkinsonism that is not responsive to levodopa (3). This statement was challenged by Zijlmans et al. in 2004 (10); in a retrospective clinicopathological study, VP subjects with vascular lesions in or near the nigrostriatal pathway showed good response to levodopa regardless of their parkinsonism onset type (acute or insidious) or their dominant clinical features (10). Since then, no other studies have aimed specifically to test levodopa response in patients with a diagnosis of VP.

An anecdotal review from 2007 reported the levodopa responsiveness in different kinds of parkinsonism. It stated that VP was responsive to levodopa in 20–40% of patients (11); however, this review was not focused exclusively on VP, and its criteria for inclusion had a VP definition that was too lax and, therefore, possibly containing a bias toward misdiagnosed VP. Recent studies have used more structured criteria to confirm diagnosis of VP (Tables 1 and 2).

**Table 2:** Winikates’ vascular parkinsonism criteria.

Winikates’ diagnostic criteria (16)
**Step 1. Parkinsonian syndrome** Presence of at least 2 of the 4 cardinal signs of parkinsonism: Tremor at rest Bradykinesia Rigidity Loss of postural reflexes.
**Step 2. Vascular score of 2 points or more** 2 points: pathologically or angiographically proven diffuse vascular disease. 1 point: onset of parkinsonism within 1 month of clinical stroke. 1 point: history of 2 or more strokes. 1 point: neuroimaging evidence of vascular disease in 2 or more vascular territories. 1 point: history of 2 or more risk factors for stroke.
Risk factors for stroke: hypertension, smoking, diabetes mellitus, hyperlipidemia, presence of heart disease associated with stroke (coronary artery disease, atrial fibrillation, congestive heart failure, valvular heart disease, mitral valve prolapse, other arrhythmias), family history of stroke, history of gout, and peripheral vascular disease

A recent cohort study on parkinsonian subjects revealed that PD subjects present a better prognosis compared to subjects with VP, who have a greater rate of institutionalization and a mortality ratio of 3 years (12). Their findings emphasize the important clinical differences and prognoses between parkinsonisms. Good levodopa response has been widely used as a prospective criteria for PD diagnosis (13), and lack of responsiveness to levodopa in a patient with parkinsonian syndrome is frequently used to pinpoint VP, even though responsiveness to levodopa is unclear in VP subjects.

This review aims to examine the clinical effects of the current pharmacological and non-pharmacological therapies for VP and to answer the following questions: (1) does available literature affirm the assertion that VP subjects are non-responders to levodopa? (2) Does nigrostriatal lesion modify the levodopa response rate in VP subjects? (3) How does VP subject’s response rate to levodopa therapy differ from PD subjects?

To answer these questions, we conducted a systematic review of available literature, looking for original articles that assessed response to different therapeutics in VP subjects. Also, we conducted a meta-analysis on the prevalence of response to levodopa therapy of VP subjects. Then, we conducted a second meta-analysis to assess if lesion of the nigrostriatal pathway affects levodopa response in VP subjects. Finally, VP subject’s levodopa response rate was compared to that of PD subjects to assess the validity of this parameter for differential diagnosis.

## Methods

### Search Strategy

**Figure 1:**
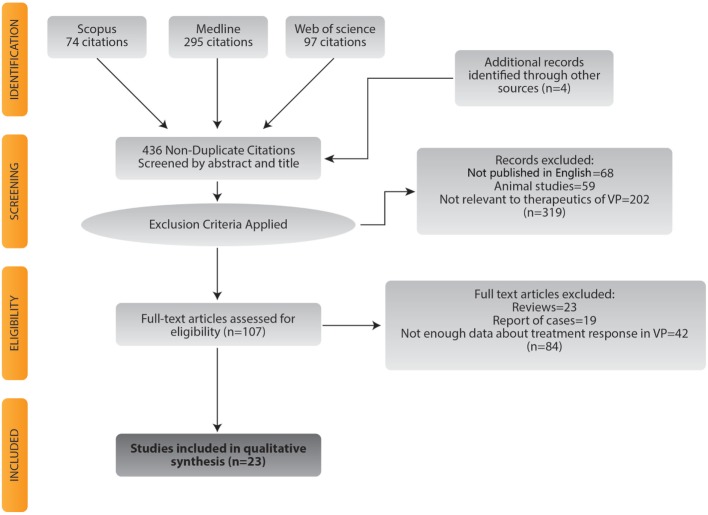
Flowchart for study selection.

In this study, we conducted a systematic review of the literature using the Preferred Reporting Items for Systematic reviews and Meta-Analyses model (14). Relevant articles were identified from MEDLINE, Scopus, and Web of Science published until June 2017. No registered clinical trials were identified from http://clinicaltrials.gov or http://clinicaltrialsregister.eu. Our search was aimed to identify studies that reported the clinical response to different kinds of therapeutic interventions in adult subjects with VP diagnosis. As historically VP has been poorly defined, we included only those studies that clearly and systematically defined VP. Given the difficulties of establishing VP diagnosis, all results and conclusions must take into account these operational definitions (Tables 1 and 2). To avoid possible bias, studies that used levodopa response as part of the definition of VP were not included. We used the following terms and Mesh terms (medical subject headings): PD, secondary; parkinsonian disorders; vascular; blood vessels; therapeutics; vitamin D; ergocalciferols; levodopa; amantadine; aripiprazole; transcranial magnetic stimulation. Full details on the search algorithm can be found in the Supplemental data. Further analysis of the references of each article was carried out to find articles that could have been excluded by the search algorithm. Only articles published in English were considered. Also, using the information retrieved by the search criteria of this systematic review, we conducted a meta-analysis focused on the prevalence of response VP subjects have to levodopa therapy. We followed the MOOSE guidelines for conducting meta-analyses of observational studies (15). Results of the search strategy are summarized in Figure 1.

### Inclusion Criteria

Studies meeting the following criteria were included in the systematic review: (1) the study’s design had to be experimental or observational (clinical trials, clinicopathological studies, cohort studies, cross-sectional observational studies, case series, and case–control studies; case reports of less than five subjects were not included in the analysis due to poor external validity of this kind of studies), (2) studies had to be explicitly focused on VP, (3) they had to report pharmacological or non-pharmacological intervention, along with the clinical response of the subjects to treatment, (4) articles had to contain a diagnosis of VP with an explicit criteria, whether known criteria [e.g., Zijlmans’ (Table 1) (1) or Winikates’ (16) (Table 2) criteria] or any other specified criteria (5) studies could not have levodopa response as part of the VP definition.

For the meta-analysis, studies were included if they met the above-mentioned criteria, but with the following substitution: (3) levodopa treatment had to be reported, along with the clinical response of the subjects [if the clinical response was measured as Unified Parkinson’s Disease Rating Scale (UPDRS) (%) reduction, then reduction had to be reported for every patient and cutoff points established to determine whether the subject was responsive or not to levodopa therapy].

### Study Selection

Two of the authors (Adán Miguel-Puga and Gabriel Villafuerte) carried out the eligibility assessment of the studies independently (15). Any discrepancy was adjudicated by consensus with a third author (Oscar Arias-Carrión). The initial evaluation of the references consisted of an analysis of the title and abstract for each screened reference. Full texts of relevant articles were then retrieved to complete the examination and eligibility process. Figure 1 shows the flowchart for study selection.

### Data Extraction

Extraction of data was carried out first by one author (Adán Miguel-Puga) and then checked by another one (Gabriel Villafuerte). The information extracted for each study included: name of first author, year of publication, continent where the study was performed, type of study design, characteristics of the study population, the diagnosis criteria for VP, the number of VP subjects (and PD if applicable) included in each study, the existence of confirmed vascular lesions and the clinical response to the intervention.

### Quality Assessment

The quality assessment was performed by two authors independently (Adán Miguel-Puga, Gabriel Villafuerte) using the STROBE checklist (17) for observational studies, which is a 22-point checklist. Any discrepancy was adjudicated by consensus with a third author (Oscar Arias-Carrión). The quality of the articles was evaluated according to the checklist. The articles were scored according to the following criteria: a point was given for every item from the checklist that was included in the study; if the item was not considered or it was impossible to determine whether it was considered or not, no point was given. A summary of the number of points obtained by each study can be found in Table 3.

**Table 3:** STROBE checklist evaluation.

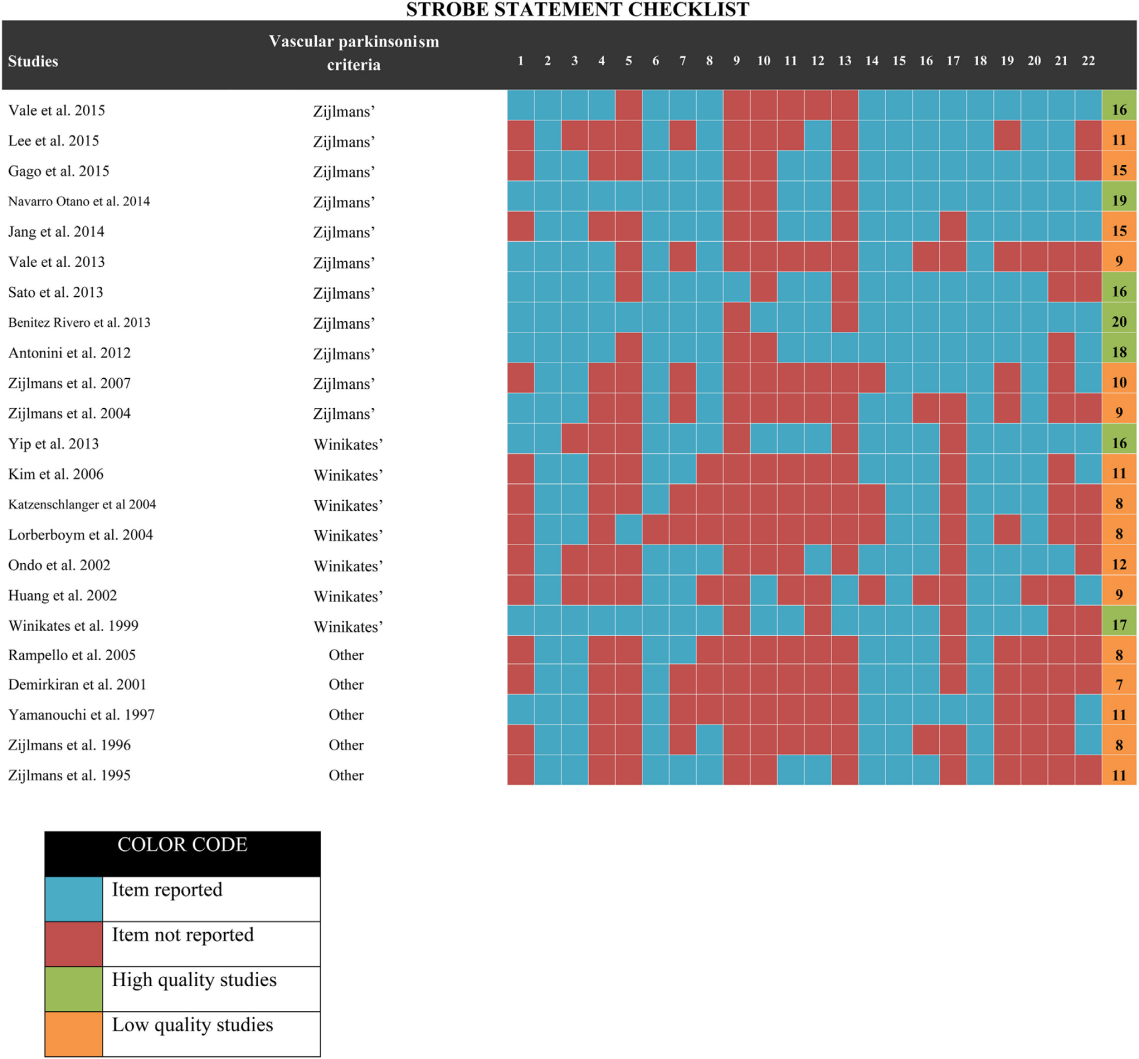

### Statistical Analysis

The statistical analysis was calculated using Comprehensive Meta-Analysis software V3. Forest plots were constructed with Graphpad Prism. Three different analyses were conducted: one to investigate the prevalence of levodopa response in VP subjects, one to assess whether the presence of nigrostriatal lesion modified the levodopa response rate in VP subjects, and one to compare the prevalence of response among VP subjects and PD subjects. To calculate the pooled effect size, both fixed and random effect models were implemented; as high heterogeneity was expected, only random effect results are reported. Fixed effect results can be consulted in the Supplementary Material. Heterogeneity was estimated using *I*^2^ and Tau^2^; *I*^2^ was computed using the fixed effect weights. Studies with *I*^2^ values from 0 to 25% were considered as having low heterogeneity, studies with values ranging from 25 to 50% were considered as having moderate heterogeneity and studies showing more than 50% were assessed as presenting high heterogeneity. To assess heterogeneity, we conducted diverse subgroup analyses to identify the origin of the heterogeneity: low vs. high-quality analysis, analysis by type of publication and analysis by continent of publication. Given the characteristics of the data extracted, no meta-regression could be done. For low- vs. high-quality analysis, we used the STROBE checklist score to divide the studies into two subjective groups (studies with 15 or less were considered as low quality, and more than 15 were considered as high quality; Table 3).

**Figure 2:**
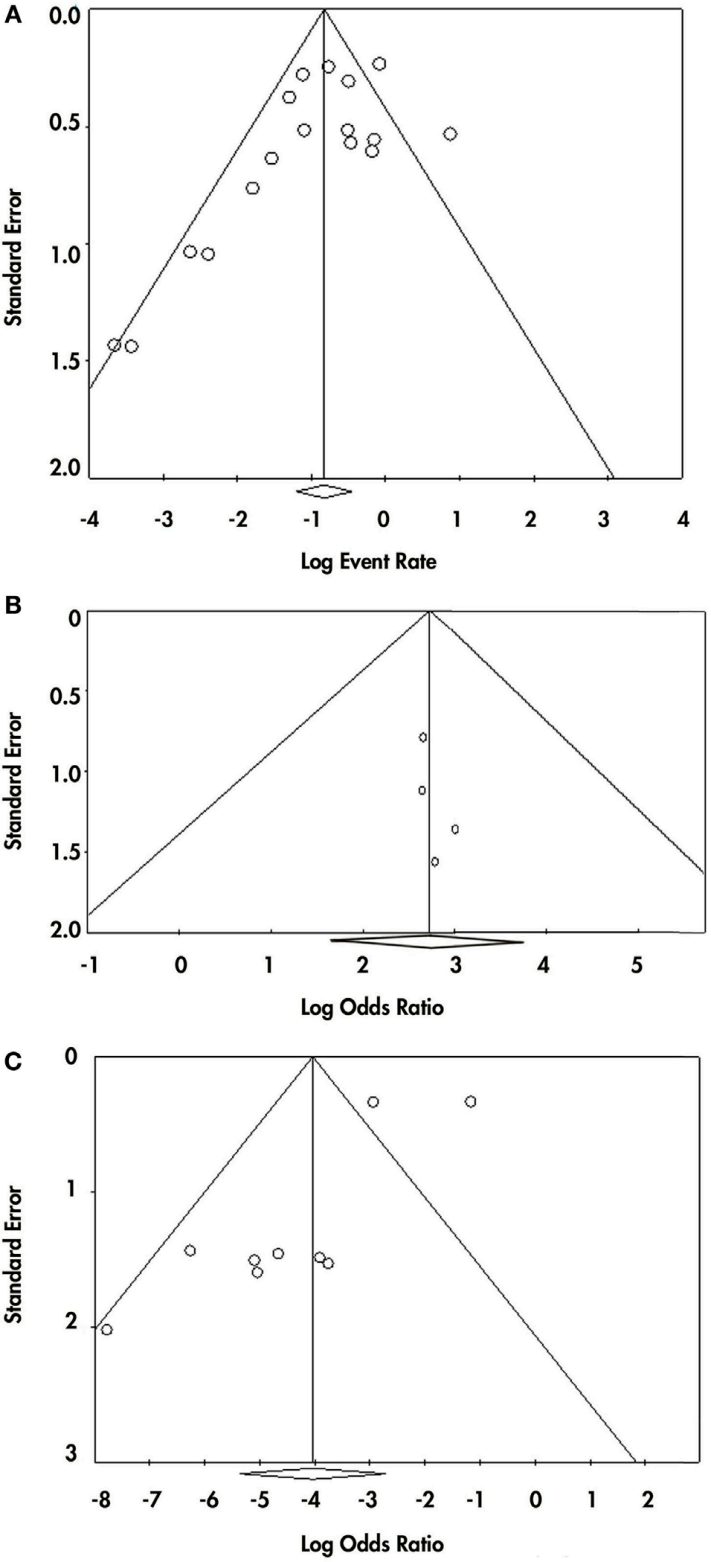
Publication bias for the three analysis made. Panel **(A)** shows the funnel plot from the event-rate analysis. Panel **(B)** shows the funnel plot from the prevalence of responsiveness in vascular parkinsonism (VP) subjects with nigrostriatal lesion vs. VP subjects without nigrostriatal lesion. Panel **(C)** shows the funnel plot from the prevalence of responsiveness in VP vs. Parkinson disease.

Because we found three different criteria that were being used for VP diagnosis, we made a subgroup analysis to investigate if the diagnostic criteria influenced the results of the overall pooled effect and heterogeneity; subgroups were combined with a random effects model. We also carried out a sensitivity analysis to estimate the influence of each study on the overall effect size; this analysis was realized by omitting one study at a time and then recalculating the effect size. Egger’s and Begg’s tests were conducted to detect possible publication bias; also, funnel plots were constructed for each of these analyses (Figure 2).

## Results

### What are the Clinical Effects of the Current Pharmacological and Non-Pharmacological Therapies for VP?

A total of 23 studies were included and analyzed in a qualitative revision. Figure 1 summarizes the study identification and selection process. Four different therapies were identified: 1 study investigated vitamin D therapy (24), 1 study assessed repetitive transcranial magnetic stimulation (rTMS) therapy (28), 1 study was focused on lumbar puncture as therapy (32) and 20 studies reported levodopa therapy (10, 16, 18–23, 25–27, 29–31, 33–38). Clinical trials were not found for any therapeutic. Pilot studies were retrieved for rTMS (28) and lumbar puncture therapy (32). A case–control study was found in the vitamin D study (24). For levodopa, we obtained 14 cross-sectional studies (16, 18–23, 29–31, 33, 35, 37, 38), 2 case–control studies (25, 27), 2 cohort studies (26, 34), and 2 clinicopathological studies (10, 36). All studies are summarized in Table 4. Specific characteristics of every study are depicted on Tables S1–S3 in Supplementary Material.

**Table 4:** Summary of included studies.

	Studies	Continent	Type of study	Treatment	Focused on treatment	Response to treatment	Comments
Zijlmans’ Criteria	Vale et al. (18)	America	Cross-sectional	Levodopa	NO	Poor	
	Lee et al. (19)	Asia	Cross-sectional	Levodopa	NO	Poor	VP subjects with nigrostriatal dopaminergic denervation had better response to levodopa
	Gago et al. (20)	Europe	Cross-sectional	Levodopa	NO	Poor	
	Navarro-Otano et al. (21)	Europe	Cross-sectional	Levodopa	NO	Poor	
	Jang et al. (22)	Asia	Cross-sectional	Levodopa	NO	Poor	
	Vale et al. (23)	America	Cross-sectional	Levodopa	NO	Poor	Poor reduction of motor UPDRS score with levodopa
	Sato et al. (24)	Asia	Case-control	Vitamin D	YES	Good	Decreased risk of falls with vitamin D
	Benitez-Rivero et al. (25)	Europe	Case-control	Levodopa	NO	Poor	
	Antonini et al. (26)	Europe	Cohort	Levodopa	NO	Poor	VP subjects with normal FP-CIT SPECT and/or LS in basal ganglia are unlikely to respond to levodopa
	Zijlmans et al. (27)	Europe	Case-control	Levodopa	NO	Poor	Poor reduction of motor UPDRS score with levodopa
	Zijlmans et al. (1, 10)	Europe	Clinicopathological	Levodopa	YES	Good	Good response to levodopa was related to lesions in or near the nigrostriatal pathway

Winikates’ Criteria	Yip et al. (28)	Asia	Pilot study	rTMS	YES	Good	VP dysfunction could be improved with rTMS
	Kim et al. (29)	Asia	Cross-sectional	Levodopa	NO	Poor	
	Katzenschlager et al. (30)	Europe	Cross-sectional	Levodopa	NO	Mixed	
	Lorberboym et al. (31)	Asia	Cross-sectional	Levodopa	NO	Poor	Normal ^123^l-FP-CIT FP-CIT SPECT may predict a poor response to levodopa
	Ondo et al. (32)	America	Pilot study	Lumbar puncture	YES	Mixed	Subjective improvement. Subjects responsive to lumbar puncture had better response to levodopa
	Huang et al. (33)	America	Cross-sectional	Levodopa	NO	Poor	
	Winikates and Jankovic (16)	America	Cross-sectional	Levodopa	NO	Poor	

Other Criteria	Rampello et al. (34)	Europe	Cohort	Levodopa	NO	Poor	
	Demirkiran et al. (35)	Europe	Cross-sectional	Levodopa	NO	Poor	
	Yamanouchi and Nagura (36)	Asia	Clinicopathological	Levodopa	NO	Poor	
	Zijlmans et al. (37)	Europe	Cross-sectional	Levodopa	NO	Poor	
	Zijlmans et al. (38)	Europe	Cross-sectional	Levodopa	NO	Poor	

*^123^l-FP-CIT SPECT, ^123^l –labeled fluoropropyl-2b-carbomethoxy-3b-(4-iodophenyl)-tropane single photon emission computed tomography; LS, lacunar strokes; rTMS, repetitive transcranial magnetic stimulation; UPDRS, Unified Parkinson’s Disease Rating Scale; VP, vascular parkinsonism (VP)*.

As the main bias in the research of VP is the criteria used to define a VP case, we divided the studies according to the diagnosis criteria utilized. The majority of the studies used either Zijlmans et al.’s criteria (1) (Table S1 in Supplementary Material) or Winikates’ criteria (16) (Table S2 in Supplementary Material). Zijlmans’ criteria was used for the diagnosis of VP in the vitamin D study (24) and in 10 of the levodopa studies (10, 18–23, 25–27) (Table S1 in Supplementary Material). Meanwhile, Winikates’ criteria were used for the diagnosis of VP in the rTMS and lumbar puncture studies (28, 32) and in five of the levodopa studies (16, 29–31, 33) (Table S2 in Supplementary Material). The studies that used neither Zijlmans’ nor Winikates’ criteria but clearly specified how the VP diagnosis was made are summarized in Table S3 in Supplementary Material, together with the criteria that were utilized in these studies (34–38).

The study on vitamin D therapy was a case–control study that was carried out on a Japanese population. It included a total of 178 subjects, 90 of them with a VP diagnosis according to Zijlmans’ criteria (1). The study evaluated the effectiveness of vitamin D therapy for prevention of falls and hip fractures. After 2 years of treatment with 1,200 UI/day of ergocalciferol, it was reported that VP subjects had 18% fewer falls compared with PD subjects (*p* < 0.001), no change in parkinsonian symptoms were observed (24).

The rTMS pilot study was carried out in Singapore; it included a total of five VP subjects. Winikates’ criteria was used for the VP diagnosis (16). The main outcomes reported were changes in the timed 10-m walk test and the score given by the UPDRS part 3. The rTMS protocol used was 20 trains of 10 s each, with 5 Hz at 80% of active motor threshold. The study showed reduced scores for the UPDRS part 3 at week 2 (*p* = 0.004), 4 (*p* = 0.022), and 6 (*p* = 0.046) and significant improvement in the timed 10-m walk test at week 2 (*p* = 0.059) and 4 (*p* = 0.026) but not at week 6 as compared to baseline (28).

Lumbar puncture therapy was carried out in a pilot study that included 40 American subjects with a VP diagnosis by Winikates’ criteria (16). 35–40 cc of cerebrospinal fluid (CSF) was drained from each subject. Out of 40 subjects, 15 showed a good subjective improvement after therapy, while the rest had mild or no improvement. The mean duration of the therapeutic response was 2.4 ± 1.2 months (32).

Finally, treatment with levodopa was evaluated in 14 cross-sectional studies (16, 18–23, 29–31, 33, 35, 37, 38), 2 case–control studies (25, 27), 2 cohort studies (26, 34), and 2 clinicopathological studies (10, 36). Except for one clinicopathological study (10), no other study was specifically aimed at testing the levodopa response of VP subjects. For the studies in which Zijlmans’ criteria was used (10, 19, 21, 22, 25–27), a total of 93 VP subjects showed a favorable response to levodopa, while 155 VP subjects showed no response (a prevalence of responsiveness in 37.5% of the subjects). On the other hand, a good response was reported in 323 PD subjects and no response in just 23 of them (90% of the subjects were responsive). In the five studies (16, 29–31, 33) that used Winikates’ criteria (16), a total of 30 subjects responded well to levodopa (a response rate of 22.05% of the subjects), while 106 subjects were non-responsive. The 5 studies with no specific criteria (34–38) showed a similar rate of response as the studies using the Winikates’ criteria: a total of 78 subjects had no response, while 25 subjects (24.27%) responded favorably to levodopa.

Four studies (18, 20, 23, 27) measured the (%) of improvement on the UPDRS part 3 of VP subjects after levodopa therapy; respective sample sizes were: 13, 17, 5, and 15 subjects; reduction of motor symptoms ranged from 5.8 to 22.25%. Two studies (18, 20) compared the UPDRS reduction to that of PD patients; VP subjects showed a reduction of 5.9–18.7% compared to 31.6–64.65% in PD subjects.

Three studies with a sample size of 20, 76, and 42 VP subjects reported that nigrostriatal dopaminergic denervation [evidenced by an abnormal fluoropropyl-2b-carbomethoxy-3b-(4-iodophenyl)-tropane single photon emission computed tomography (FP-CIT SPECT)] may predict a favorable response to levodopa in VP subjects (19, 26, 31).

### Does Available Literature Affirm the Assertion that VP Subjects are Non-Responders to Levodopa?

To determine the prevalence of favorable response to levodopa therapy among VP subjects, a meta-analysis of the data was conducted.

A total of 17 studies were included in the meta-analysis. For this analysis, the following studies are summarized; 13 cross-sectional studies (10, 16, 19, 21, 22, 29–31, 33, 35–38), 2 case–control studies (25, 27), 2 cohort studies (26, 34) (clinicopathological studies were considered as cross-sectional studies). Of these studies, 2 studies were conducted in America (16, 33), 10 in Europe (10, 21, 25–27, 30, 34, 35, 37, 38), and 5 in Asia (19, 22, 29, 31, 36). All studies included both male and female subjects, but the response to levodopa was not divided by sex, so this variable could not be included in the meta-analysis. According to the year of publication, 13 studies were published after the year 2000 (10, 19, 21, 22, 25–27, 29–31, 33–35), while the remaining 4 studies were published in the year 2000 or earlier (16, 36–38). The estimated quality of all included studies was in the range of 7–20 points on the STROBE checklist (17). These ratings have been reported in Table 3.

**Figure 3:**
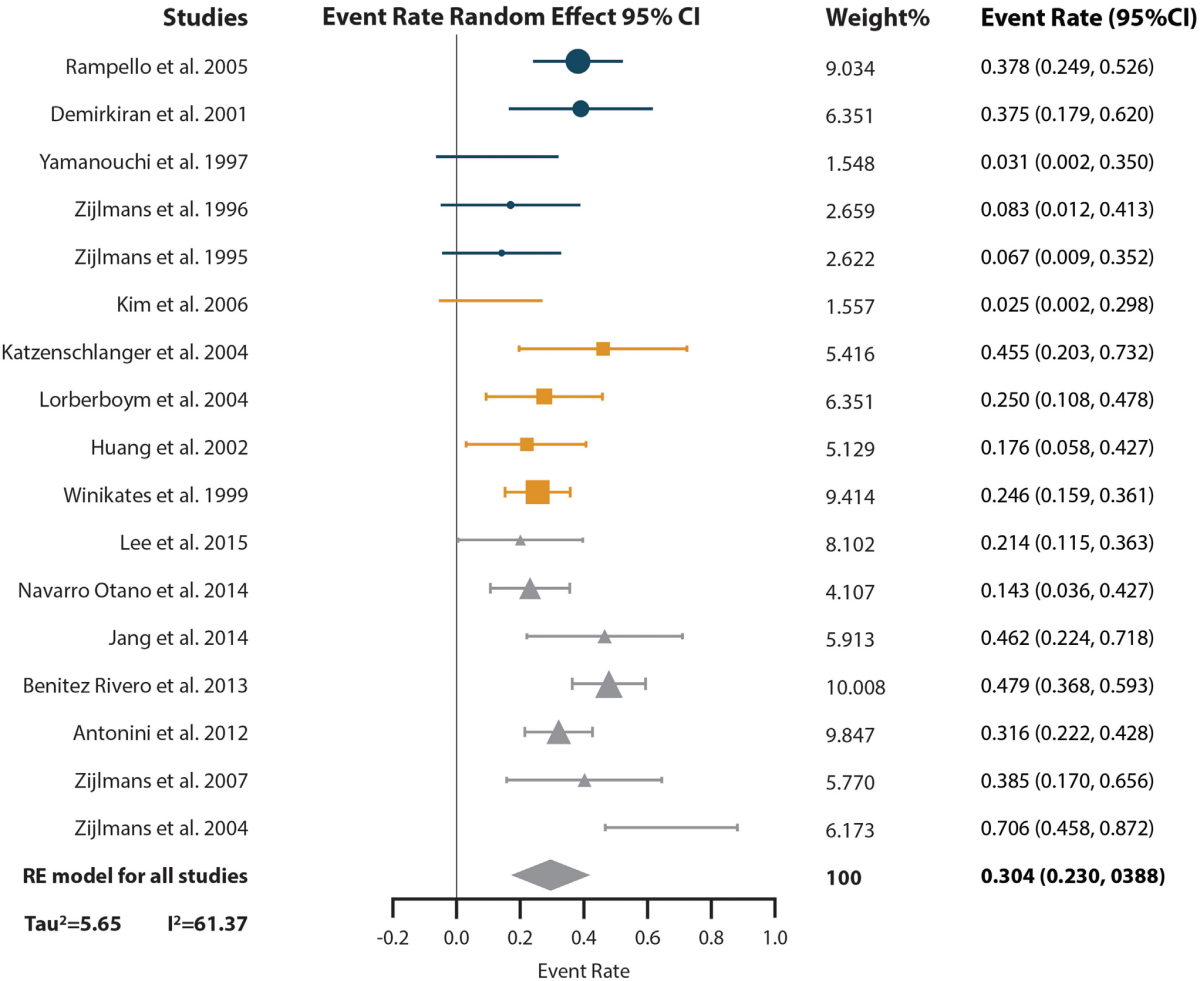
Pooled random effect event rate and 95% confidence interval (CI) for the prevalence of levodopa response in vascular parkinsonism subjects. Circles represent studies with the “Other” diagnostic criteria, squares represent studies with the “Winikates’” diagnostic criteria, and triangles represent “Zijlmans’” diagnostic criteria. Size of the geometrical figures is proportional to their respective relative weight. RE, random effect.

The results of the event-rate meta-analysis of the prevalence of levodopa response in subjects with VP are reported in Figure 3. The levodopa response of a total of 487 VP subjects distributed in 17 studies was included in this analysis. The overall event rate found was of 0.304 [95% confidence intervals (CI) of 0.230–0.388]. With the subgroup analysis, we found that the event rate changed depending on the diagnostic criteria; however, CI 95% overlapped (Supplementary Material). Sensitivity analysis showed event rates from 0.287 (CI 95% 0.234–0.402) to 0.316 (CI 95% 0.241–0.400) (Supplementary Material). High heterogeneity was found (*I*^2^ = 61.37% and Tau^2^ = 5.65). The subgroup analysis showed that all the VP diagnosis criteria had heterogeneity (*I*^2^ “Zijlmans’” = 60.5%, *I*^2^ “Winikates’” = 33.153%, *I*^2^ “Other” = 68.11%) and that quality of the studies did not influence heterogeneity (*I*^2^ high-quality studies = 73.12% and *I*^2^ low-quality studies = 60.06%) (Supplementary Material). Changes in heterogeneity distribution were found in the subgroup analysis for continent and type of study. Continent subgroup analysis showed that American studies had no heterogeneity (*I*^2^ = 0%), while the heterogeneity concentrated in Asian and European studies was high (*I*^2^ = 55.44% and *I*^2^ = 58.43%, respectively) (Supplementary Material). Type of study subgroup analysis indicated that case and control studies and cohort studies had no heterogeneity (both with *I*^2^ = 0%), while the heterogeneity was produced by the cross-sectional studies (*I*^2^ = 60.87%) (Supplementary Material). Information of sensitivity and group subanalysis data are depicted on Tables S4–S8 in Supplementary Material.

Visual inspection of funnel plot showed asymmetry in the inferior part of the plot, so publication bias remains a possibility (Begg test *p*-value = 0.02 and Egger test *p*-value = 0.05) for this analysis (Figure 2A).

### Does Nigrostriatal Lesion Modify the Levodopa Response Rate in VP Subjects?

**Figure 4:**
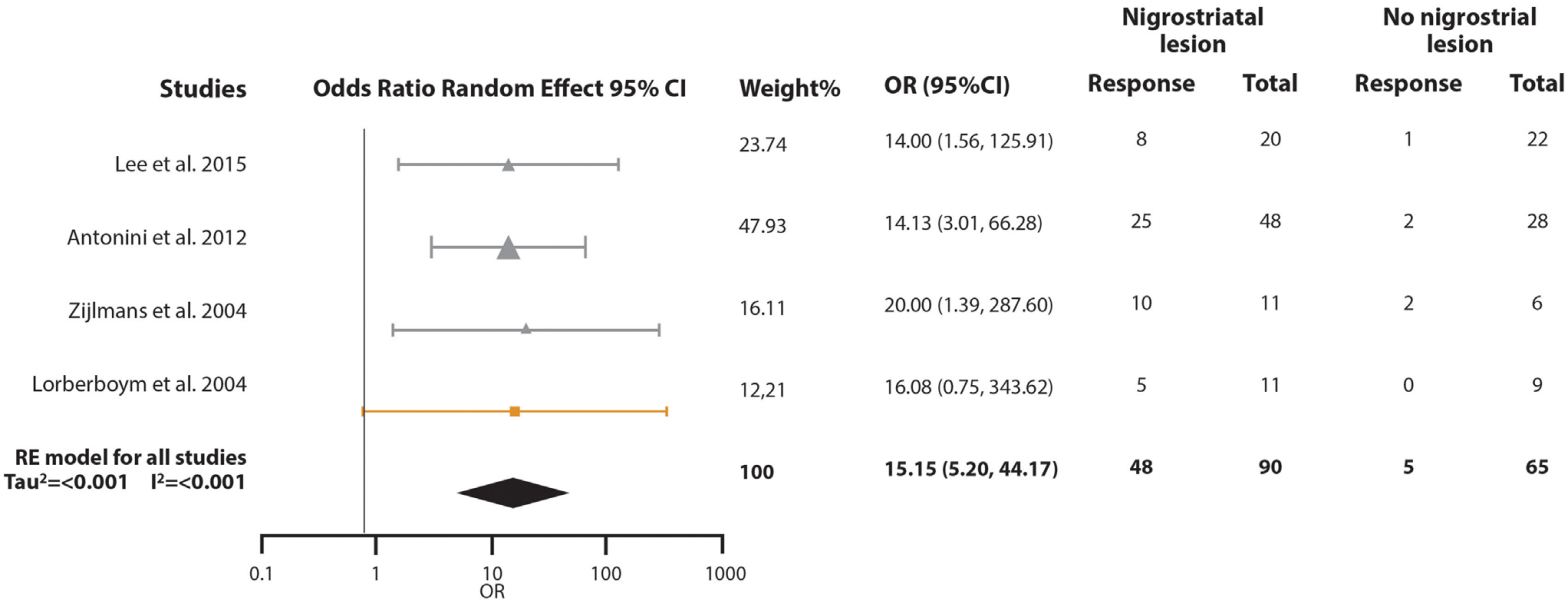
Pooled odds ratio (OR) and 95% confidence interval (CI) for the probability of vascular parkinsonism (VP) subjects responsiveness to levodopa with nigrostriatal lesion compared with VP subjects without nigrostriatal lesion. Circles represent studies with the “Other” diagnostic criteria, squares represent studies with the “Winikates’” diagnostic criteria, and triangles represent “Ziljmans’” diagnostic criteria. Size of the geometrical figures is proportional to their respective relative weight. RE, random effect.

For the second meta-analysis, we pooled the odds ratio (OR) of the probability of responding to levodopa in VP subjects with nigrostriatal lesion compared with VP subjects without nigrostriatal lesion (Figure 4). A total of 155 VP subjects were included: 90 with nigrostriatal lesion and 65 without lesion. The subjects were distributed in four studies. The pooled OR showed that VP subjects with nigrostriatal lesion are much more likely to respond to levodopa (OR = 15.148, 95% CI 5.195–44.169). Minimal heterogeneity is inferred in this analysis as demonstrated by an *I*^2^ = 0% and a Tau^2^ = 0. Due to the lack of heterogeneity, fixed and random effect, pooled effect and relative weights were the same. No subgroup analysis was performed due to lack of heterogeneity. Sensitivity analysis did not change the tendency in the effect as it had an OR ranging from 14.36 (95% CI 4.46–46.19) to 16.14 (95% CI 3.66–71.16) (Supplementary Material). Funnel plot inspection did not demonstrate asymmetry and the Egger and Begg *p*-values were above 0.05 (Figure 2B).

### How does VP Subject’s Response Rate to Levodopa Therapy Differ from Parkinson’s Disease Subjects?

**Figure 5:**
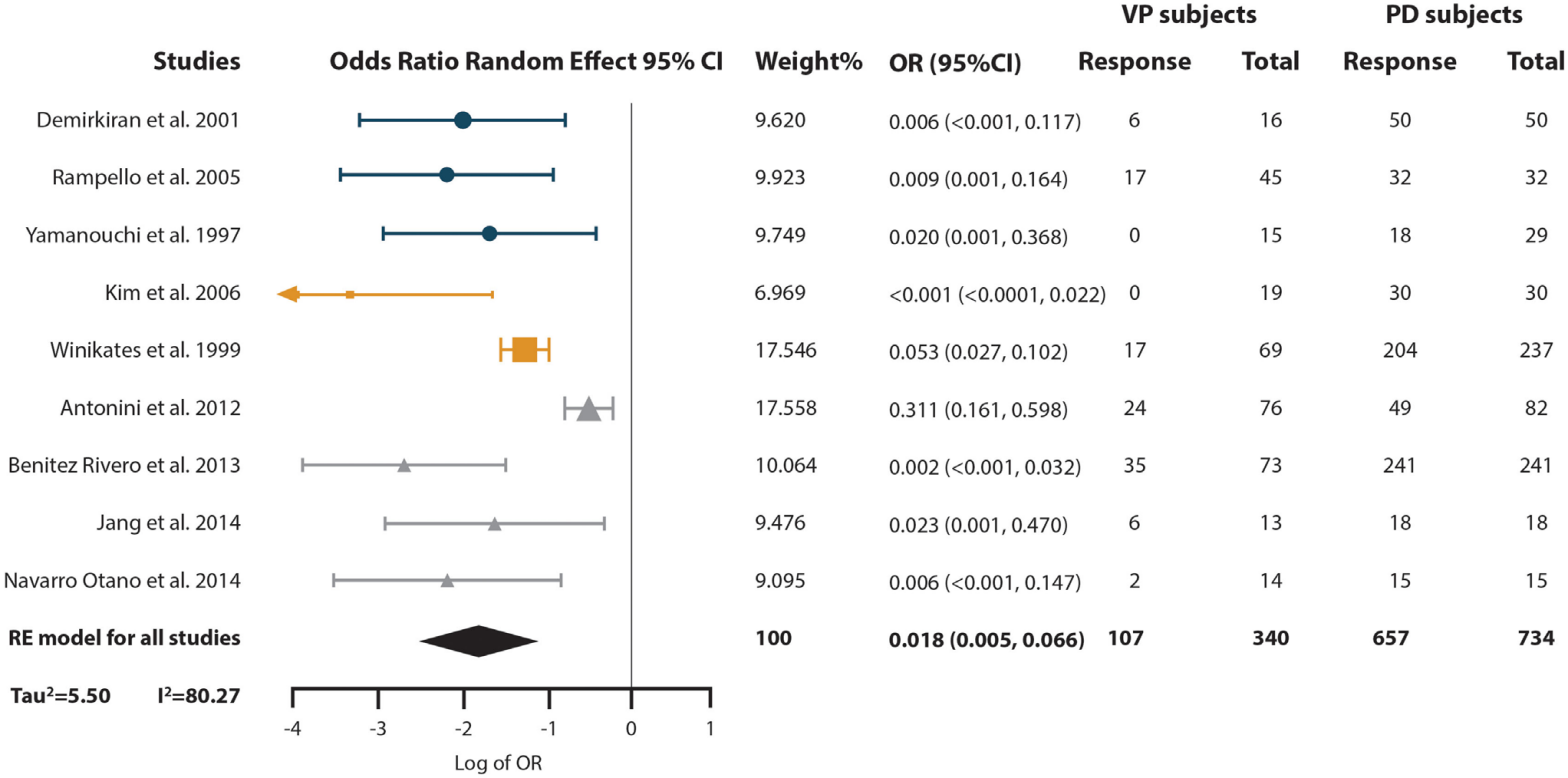
Pooled odds ratio (OR) and 95% confidence interval (CI) for the probability of vascular parkinsonism (VP) subjects responsiveness to levodopa compared with the probability of PD subjects response to it. Circles represent studies with the “Other” diagnostic criteria, squares represent studies with the “Winikates’” diagnostic criteria, and triangles represent “Zijlmans’” diagnostic criteria. Size of the geometrical figures is proportional to their respective relative weight. RE, random effect.

To answer this question, we performed a third meta-analysis where we pooled the OR of the probability VP subjects have of responding to levodopa compared with the probability of PD subjects (Figure 5). The response of a total of 340 VP subjects and 734 PD subjects distributed in nine studies were included in this analysis. An overall OR of 0.018 (CI 95% 0.005–0.066) was found. A high heterogeneity measure was found (*I*^2^ = 80.27% and Tau^2^ = 2.50). The subgroup analysis by VP diagnosis criteria also evidenced different ORs and 95% CI, although the CIs overlapped (Supplementary Material). This same subgroup analysis by VP diagnosis criteria exhibited no heterogeneity in the “Other” subgroup (*I*^2^ = 0%), while great heterogeneity in the “Zijlmans’” and “Winikates’” subgroup was found (*I*^2^ = 84.06 and 82.04%, respectively) (Supplementary Material). Surprisingly, subgroup analysis for quality showed null heterogeneity in the low-quality group (*I*^2^ = 0%) whereas high heterogeneity was found in the high-quality group (*I*^2^ = 88.25%) (Supplementary Material). Sensitivity analysis pointed out ORs ranging from 0.011 (95% CI 0.003–0.038) to 0.024 (95% CI 0.007–0.089) Supplementary Material (para unificarlo con todos). Funnel plot showed asymmetry, especially in the upper part of the graph and statistical analysis for publication bias showed evidence of this bias (Begg test *p*-value = 0.67 and Egger test *p*-value = 0.01); however, results remain solid due to a “classic fail-safe N”: the calculated number of studies missing needed to bring the *p*-value greater than 0.05 would be 315 (Figure 2C). Information of sensitivity and group subanalysis data are depicted on Tables S9–S14 in Supplementary Material.

## Discussion

Our systematic review of the available literature revealed that few studies had been done on potential therapeutics for VP. Also, the evidence retrieved of the proposed VP therapies comes from observational studies and not from prospective and controlled studies.

Subjects treated with rTMS showed clinical improvement as validated by the timed 10-m walk test and UPDRS part 3 (28). However, the study had no sham control and therefore no blindness; results could still be adjudicated to a placebo effect. The sample size is a major limitation of this study; only 5 VP subjects were examined; therefore external validity is extremely low (28). Jang et al. (22), in a randomized, double-blind sham-controlled study on 20 subjects with parkinsonism, showed that 10 Hz rTMS over M1 and dorsolateral prefrontal cortex could be effective for freezing of gait (a pivot symptom of VP). However, results from their study cannot be applied to VP subjects as the VP diagnosis criterion from this study was not explicitly stated, and therefore, the probability of misdiagnosis remains high. Our systematic review found another study (39) reporting treatment of VP with rTMS that showed promising results but ultimately it was not included in the analysis as the VP definition the authors used excluded subjects with good response to levodopa (40).

As for lumbar puncture therapy, a recent review by Korczyn (3) stated that “CSF drainage to treat patients with VP has produced positive results”; however, based on our systematic review only one study has explored the effect of lumbar puncture on VP (32). Previously reported studies focused on idiopathic normal pressure hydrocephalus (iNPH) (41–43), and while these two disorders share certain symptoms and radiological signs, VP has a different etiology and pathophysiology (42). Vizcarra et al. (6) pointed out that no clinical or radiological feature can accurately differentiate VP from iNPH. To evaluate if VP is responsive to lumbar puncture, pathological corroboration of vascular disease would require knowledge of the positive and negative predictive values of any method proposed to differentiate VP from iNPH in order to make clinical judgments as to potential treatments.

During the 2-year follow-up period of an open-label study, vitamin D (at a daily dose of 1,200 IU ergocalciferol) was proven to reduce the number of falls and hip fractures in VP patients as compared to those with PD. No potential mechanisms of action were explored, but vitamin D is theorized to play an active role in muscle strength. Limitations of the study include an absence of a placebo and age-matched controls. Due to methodological constraints, this study is not enough to confirm the effects of vitamin D on VP subjects. If more evidence is documented, vitamin D could be recommended as an adjuvant therapy to prevent complications of VP (24).

Medication used for secondary stroke prevention may be a suitable option for preventing the worsening of VP symptoms and improving its prognosis as they help to control vascular risk factors (3, 44). However, none of the studies included in our systematic review contained information on subjects taking medication for secondary stroke prevention or its influence on clinical response. To the best of our knowledge, there have been no clinical trials or cohort studies that focus on this issue in VP subjects. However, in the absence of definitive prospective controlled studies, it may be reasonable to extrapolate from studies and clinical results of treatment of stroke in general.

As previously stated, levodopa therapy is the most effective treatment for PD. According to our systematic review, it is also the most studied option for VP therapy. However, studies have determined that levodopa is a non-effective therapy (3) for VP subjects. This conclusion has been perpetuated by classic studies (45, 46) in which VP was poorly defined or not defined at all. Our meta-analysis revealed that approximately 30% of VP subjects do respond to levodopa therapy. We found high heterogeneity and initially guessed that this heterogeneity would present a problem due to the different criteria used for diagnosis in each study. However, surprisingly, heterogeneity was not explained by the diagnosis criteria but by the type of study. Regardless of the diagnostic criteria reported, the event-rate meta-analysis showed a low proportion of subjects responding to levodopa. Subjects diagnosed with Zijlmans’ criteria showed the highest proportion of response to levodopa (OR 0.379, CI 95% 0.262–0.513; Supplementary Material); however, due to overlapping CI, no statistical difference was observed in comparison to other diagnostic criteria. Although our results show that the criteria used for diagnosis do not change the event-rate response, we highly recommend maintaining the actual tendency of diagnosing VP subjects with standardized criteria until more definitive knowledge is obtained. This would increase the external validity of future research and certainly will make results between studies more comparable.

In the revised studies, vascular lesions were found on external capsule, corona radiata, thalamus, pons, basal ganglia, and substantia nigra (Tables S1 and S2 in Supplementary Material). We decided to pool single photon emission computed tomography (SPECT) studies with the clinicopathological study in which nigrostriatal lesion was confirmed. Even though two different methods of assessing nigrostriatal integrity were used, sensitive analysis showed that excluding the clinicopathological study did not change the outcome. Our results showed that VP subjects with lesion in the nigrostriatal pathway are 15 times more likely to respond to levodopa than VP subjects without these lesions. Although the OR is large, we should still take into account that the proportion of the VP subjects with nigrostriatal lesion that respond to levodopa is just above 50%.

Previously, Vizcarra et al. reported that for a true VP diagnosis, ischemic or hemorrhagic lesions in the nigrostriatal pathway were needed (6). Otherwise, diagnoses made by clinical presentation and magnetic resonance imaging may have a certain degree of inaccuracy. Our results concur with Vizcarra et al., a dopamine transporter deficiency measured with SPECT, predicts a much better response to levodopa therapy, and therefore dopamine transporter deficiency can be a good predictor of levodopa response in VP patients. However, the negative predictive value cannot be ascertained. Consequently, the process of clinical diagnosis and determination of treatment also has to consider the consequences of failure to use levodopa due to a false negative study.

Finally, we found that the probability of responsiveness to levodopa for a VP subject is 0.018 times the probability of a PD subject. This result could also be expressed in the following way: for every 55 PD subjects that respond to levodopa only one VP subject responds to it. This disparity in responsiveness can be useful in the differential diagnosis of VP and PD, but should be read with caution: even when VP subjects’ probability of responding is very low compared to PD subjects, approximately 30% of the VP subjects do respond to levodopa. The last statement should discourage movement disorder specialists from using lack of levodopa response to pinpoint VP. Interestingly, this disparity may suggest different pathophysiological mechanisms underlying the clinical phenomena of VP, particularly as it related to the presence and absence of nigral involvement, compared to idiopathic PD. It is important to highlight that vascular risk factors are more prevalent in the aging population. As a great proportion of PD subjects have vascular risk factors and even radiological evidence of lacunar strokes (4), part of making a correct differential diagnosis between VP and PD should be an assessment of the causal role of the vascular lesions in the clinical syndrome.

Our study has several limitations: first, only observational studies were found, so potential bias and inaccurate conclusions are possible concerning the efficacy of treatments; also, most of the studies retrieved had a small sample size (14–47 subjects per study); therefore, inherent bias may be implicit. Second, we did not search for unpublished data and only papers published in English were considered; therefore, publication bias cannot be ruled out. Third, as not all studies reported mean equivalent doses of medication, a further meta-regression analysis of dose–response could not be done; consequently, we could not assess if dosage has any influence on clinical response. Fourth, although it has been reported that VP subjects on average have a greater age than PD subjects (16, 21, 22, 29, 35), most studies included in this analysis do not report the specific age of the responsive subjects. As with age, genre differences in levodopa responsiveness have been reported in studies (16, 20, 25). However, the specific genre of responsive subjects was not reported in the studies that met the criteria of this review; thus it could not be analyzed if a subject’s age or genre has any interaction with clinical response rates of treatment. Finally, even with standardized criteria as those of Ziljmans’ and Winikates’, the diagnosis of VP is still inexact; its true prevalence and incidence are unknown; therefore, any recommendations given in this review must take into account the limitations of VP diagnosis. To the best of our knowledge, no study has aimed to measure the positive and negative predictive values of standardized criteria.

We conclude that further investigation of diagnostic procedures is needed to provide positive and negative predictive values for this neurological disorder. Additionally, randomized placebo-controlled clinical trials of available therapeutic interventions using a clear definition of VP are urgently needed to be able to provide optimal care for VP subjects and avoid the consequences of false positive and false negative diagnoses. Although to date there is insufficient evidence in the literature to make any recommendations as to the treatments for VP, the small number of subjects that are responsive to levodopa certainly merits a trial use of this drug to ascertain individual responsiveness. Also, adjuvant therapy with vitamin D and rTMS may be promising. Despite the limitations of differing diagnostic criteria, the results of this meta-analysis would seem to indicate that responsiveness to levodopa is not a reliable determinant for a differential VP/PD diagnosis.

## Author Contributions

AM-P, GV, JS-P, and OA-C were involved in conceptualization, design, and interpretation of results. AM-P and GV were in charge of coding, data extraction, and statistical analysis. OA-C: data output analysis. All four authors wrote and approved this manuscript.

## Conflict of Interest Statement

The authors declare that the research was conducted in the absence of any commercial or financial relationships that could be construed as a potential conflict of interest.
