# Supplementary material for: Therapeutic Interventions for Vascular Parkinsonism: A Systematic Review and Meta-analysis
Source: Front Neurol. 2017 Sep 22;8:481. doi: 10.3389/fneur.2017.00481 (PMC5614922; doi:10.3389/fneur.2017.00481)
Supplement: Supplementary file 1 [file data_sheet_1.docx]

Supplementary Material

**Therapeutic Interventions for Vascular Parkinsonism:**

**A systematic review and meta-analysis**

**Adán Miguel-Puga^1,2^, Gabriel Villafuerte^1,2^, José Salas-Pacheco^3^, Oscar Arias-Carrión^1,4*^**

^1^Unidad de Trastornos del Movimiento y Sueño (TMS, Hospital General Dr. Manuel Gea González. Ciudad de México, México.

^2^Plan de Estudios Combinados en Medicina (PECEM), Facultad de Medicina, Universidad Nacional Autónoma de México. Ciudad de México, México.

^3^Instituto de Investigación Científica, Universidad Juárez del Estado de Durango. Durango, México.

^4^Centro de Innovación Médica Aplicada (CIMA), Hospital General Dr. Manuel Gea González, Ciudad de México, México.

***Correspondence:**Oscar Arias-Carrión MD, PhD, Unidad de Trastornos del Movimiento y Sueño, Hospital General Dr. Manuel Gea González/IFC-UNAM, Calzada de Tlalpan 4800, Delegación Tlalpan, 14080 Mexico City, Mexico. Tel: 4000 3000 ext 4504

**Pubmed Search algorithm.**

((("parkinson’s disease, secondary"[MeSH Terms] OR ("parkinson’s"[All Fields] AND "disease"[All Fields] AND "secondary"[All Fields]) OR "secondary parkinson’s disease"[All Fields] OR "parkinson’s disease, secondary"[All Fields]) AND ("blood vessels"[MeSH Terms] OR ("blood"[All Fields] AND "vessels"[All Fields]) OR "blood vessels"[All Fields] OR "vascular"[All Fields])) OR (("blood vessels"[MeSH Terms] OR ("blood"[All Fields] AND "vessels"[All Fields]) OR "blood vessels"[All Fields] OR "vascular"[All Fields]) AND ("parkinsonian disorders"[MeSH Terms] OR ("parkinsonian"[All Fields] AND "disorders"[All Fields]) OR "parkinsonian disorders"[All Fields] OR "parkinsonism"[All Fields]))) AND ((Therapeutics) OR ("vitamin D"[MeSH Terms] OR "vitamin D"[All Fields] OR "ergocalciferols"[MeSH Terms] OR "ergocalciferols"[All Fields]) OR ("levodopa"[MeSH Terms] OR "levodopa"[All Fields]) OR ("amantadine"[MeSH Terms] OR "amantadine"[All Fields]) OR ("aripiprazole" [All fields]) OR ("Transcranial Magnetic Stimulation"[All fields] OR "Transcranial Magnetic Stimulation"[Mesh])) NOT (("alzheimer’s disease"[MeSH Terms] OR ("alzheimer’s"[All Fields] AND "disease"[All Fields]) OR "alzheimer’s disease"[All Fields]) OR ("mptp poisoning"[MeSH Terms] OR ("mptp"[All Fields] AND "poisoning"[All Fields]) OR "mptp poisoning"[All Fields]) OR ("Manganese Poisoning"[Mesh] OR "Manganese Poisoning"[All Fields] OR ("manganese"[All Fields] AND "poisoning"[All Fields])) OR ("parkinson’s disease, postencephalitic"[MeSH Terms] OR ("parkinson’s"[All Fields] AND "disease"[All Fields] AND "postencephalitic"[All Fields]) OR "postencephalitic parkinson’s disease"[All Fields])).

**Table S1. VP studies with Zijlmans´ criteria diagnosis.**

| **Author & country** | **Type of study** | **Treatment (duration for VP)** | **Population** | **Vascular lesions** | **Purpose of the study** | **Results for VP and treatment** | **Conclusion for VP and treatment** | |
| --- | --- | --- | --- | --- | --- | --- | --- | --- |
| Vale et al., (2015) (18).  Brazil. | Cross- sectional. | ***Levodopa (2.8±3.1 years).*** | 45 subjects: 15 VP and 30 PD. | LS in SNpc 1/15, Multiple LS 10/15, P&DSWML 4/15. | To compare the clinical and radiological features of VP and PD. | Mean motor UPDRS (%) reduction after levodopa was 5.9±4.6 % in VP subjects and 31.6±11.5 in PD subjects. | VP subjects had poor motor benefit from levodopa compared with PD subjects. | |
| Lee et al., (2015)(19).  Republic of Korea. | Cross- sectional. | ***Levodopa (not reported).*** | 71 subjects: 42 VP and 30 29 HS. | WML in lobar, subcortical or periventricular regions. Subjects with lesions in BG, thalamus, brainstem or cerebellum were excluded. | To study the prevalence of coexisting nigrostriatal dopaminergic denervation and associated clinical features in VP. | 33/42 subjects had poor or partial response to levodopa. 9/42 VP subjects had good response to levodopa. Significant difference in good response for levodopa in VP with nigrostriatal dopaminergic denervation (40%) vs VP without nigrostriatal dopaminergic denervation (4.5%) | VP subjects with nigrostriatal dopaminergic denervation had better response to levodopa than VP subjects without nigrostriatal dopaminergic denervation. | |
| Gago el al., (2015) (20).  Portugal. | Cross- sectional. | ***Levodopa (not reported).*** | 15 subjects: 5 VP and 10 PD. | Subcortical WML or BG lesions. | To characterize postural stability in PD and VP subjects and analyze the response to levodopa using wearable inertial sensors. | Mean motor UPDRS (%) reduction after levodopa was 19 % in VP subjects and 57.5% in PD subjects. | VP had poor motor benefit from levodopa compared with PD subjects. Levodopa did not have a positive effect of the range of anterior-posterior postural sway in VP subjects. | |
| Navarro-Otano et al. (2014) (21).  Spain. | Cross- sectional. | ***Levodopa (not reported).*** | 39 subjects: 15 VP, 15 PD and 9 HS. | P&DSWML; LS in thalamus, globus pallidus and frontal lobes. | To analyze the usefulness of ^123^I-MIBG cardiac imaging, UPSIT score and ^123^I-FP-CIT SPECT in the differential diagnosis between VP and PD | A total of 2/15 (14.3%) VP subjects showed a good response lo levodopa while 15/15 PD subjects showed good response lo levodopa. | VP subjects had poor motor benefit from levodopa compared with PD subjects. | |
| Jang et al (2014) (22).  Republic of Korea. | Cross- sectional. | ***Levodopa (not reported).*** | 41 subjects: 13 VP, 18 PD and 10 HS. | P&DSWML, LS & juxtaventricular and juxtacortical lesions. | To evaluate VP subjects using triple stimulation technique and determine if it is effective for differentiating VP from PD. | A total of 6/13 of VP subjects showed a good response lo levodopa while 18/18 PD subjects showed good response lo levodopa. | VP subjects had poor motor benefit from levodopa compared with PD subjects. | |
| Vale et al., (2013) (23).  Brazil. | Cross-sectional. | ***Levodopa (2.9 years)*** | 17 VP subjects. | LS in SNpc 1/17, periventricular WML 2/17, multiple LS 10/17, P&DSWML 4/17. | To report the clinical and neuroimaging findings in VP. | A mean 5.8±4.4 points reduction in the motor UPDRS scale after levodopa. | VP subjects had a poor reduction of motor UPDRS score with levodopa. | |
| Sato et al., (2013) (24).  Japan. | Cases & Controls. | ***Vitamin D (2 years)***. | 178 subjects: 90 VP, 88 PD. | Not specified. | To evaluate the effectiveness of vitamin D for the prevention of falls and hip fractures in VP and PD subjects. | Falls in VP subjects were reduced after two years of treatment (from 34% to 16%). Only 1 hip fracture in the VP group and 7 hip fractures in the PD group. | Vitamin D supplementation in VP subjects decreased risk of falls. | |
| Benitez-Rivero et al., (2013) (25).  Spain. | Cases & Controls. | ***Levodopa (not reported).*** | 386 subjects: 106 VP, 280 PD. | P&DSWML 95/106, LS 83/106 & territorial infarction 16/106. | To analyze the differences in the clinical features and ^123^l-FP-CIT SPECT imaging among VP and PD subjects. | 76 VP subjects used levodopa and only 35 were responsive to treatment (47.9 %) while all PD subjects responded to treatment (100%) | VP subjects had poor motor benefit from levodopa compared with PD subjects. | |
| Antonini et al., (2012) (26)  Italy | Cross-sectional cohort. | ***Levodopa (not reported).*** | 158 subjects: 76 VP, 82 PD. | WML in lobar sites, deep subcortical WML, LS in BG, thalamus and other infratentorial regions. | To differentiate PD from VP by imaging techniques and to assess the relationship between clinical features, presynaptic dopamine function and topography of WML. | 52/76 (68.4%) of VP subjects had negative levodopa response, while only 33/82 (40%) PD subjects had negative response to levodopa. 93% of VP subjects with normal FP-CIT SPECT had poor response to levodopa. | VP subjects had poor motor benefit from levodopa compared with PD subjects.  VP subjects with normal FP-CIT SPECT and/or LS in basal ganglia are unlikely to respond to levodopa. | |
| Zijlmans et al., (2007) (27).  United Kingdom. | Cases & Controls. | ***Levodopa (4.5 years).*** | 41 subjects: 13 VP, 14 PD, 14 HS. | LS in SN 1/13, globus pallidus/putamen 7/13, P&DSWML 12/13. | To compare the presynaptic dopamine function between PD and VP. | Mean motor UPDRS (%) reduction after levodopa was 14%. Presynaptic dopamine function was not significantly correlated with the change of UPDRS (%) after levodopa. | VP subjects had a poor reduction of motor UPDRS score with levodopa. | |
| Zijlmans et al., (2004) (10).  United Kingdom. | Clinicopathological. | ***Levodopa (not reported).*** | 17 VP subjects. | LS in substantia nigra, putamen, caudate nucleus and globus pallidus. | To correlate a positive response to levodopa in VP subjects with the presence of nigrostriatal vascular pathology. | 3/17 VP subjects showed excellent response, 9/17 showed good response, 2/17 showed moderate response and 3/17 showed no response to levodopa therapy. | Good response to levodopa was related to lesions in or near the nigrostriatal pathway. | |
| 123l –labelled fluoropropyl-2b-carbomethoxy-3b-(4-iodophenyl)-tropane single photon emission computed tomography (123l-FP-CIT SPECT), 123I-meta-iodobenzylguanidine (123I-MIBG ), basal ganglia (BG), healthy subjects (HS), lacunar strokes (LS), Parkinson´s disease (PD), periventricular & deep subcortical white matter lesions (P&DSWML), substantia nigra pars compacta (SNpc), Unified Parkinson´s Disease Rating Scale (UPDRS), University of Pennsylvania Smell Identification Test (UPSIT), vascular parkinsonism (VP), white matter lesions (WML). | | | | | | | |  |

| **Author & country** | **Type of study** | **Treatment (duration for VP)** | **Population** | **Vascular lesions** | **Purpose of the study** | **Results for VP and treatment** | **Conclusion for VP and treatment** |
| --- | --- | --- | --- | --- | --- | --- | --- |
| Yip et al., (2013) (28).  Singapore. | Pilot study. | ***rTMS (5 days)***. | 5 VP subjects. | P&DSWML, LS in external capsule, corona radiata, caudate, thalamus, pons and lentiform nuclei. | To explore rTMS as a potential new and safe therapy for VP. | Improved time in the T10MW at 4 weeks post-rTMS, but not at 6 weeks. Reduced scores in the UPDRS at weeks 2, 4 and 6. | VP dysfunction could be improved with rTMS. |
| Kim et al., (2006) (29).  Republic of Korea. | Cross- sectional. | ***Levodopa (not reported).*** | 65 subjects: 19 VP, 30 PD and 16 HS. | P&DSWML and LS in BG. | To evaluate the role of cardiac MIBG scintigraphy in the diagnosis of VP. | None of the 19 VP subjects showed good long term response to levodopa, while all the PD subjects showed good long term response to levodopa. | VP subjects had a poor response to levodopa compared with PD subjects. |
| Katzenschlanger et al., (2004) (30).  United Kingdom. | Cross- sectional. | ***Levodopa (not reported).*** | 59 subjects: 14 VP, 18 PD and 27 HS. | 12/14 P&DSWML , 7/14 LS in BG, 1/14 LS in substantia nigra, 1/14 bilateral midbrain lesions. | To assess olfactory function in subjects with VP compared to those with PD and HS. | 6/11 VP subjects had a subjective poor or equivocal response to levodopa, 5/11 VP subjects had a good or moderate response to levodopa. | VP subjects had a mixed response to levodopa. |
| Lorberboym et al., (2004) (31).  Israel. | Cross- sectional. | ***Levodopa (not reported).*** | 30 subjects: 20 VP and 10 HS. | 10/20 LS and periventricular WML, 7/20 LS, 3/20 territorial stroke. | To evaluate the striatal uptake of ^123^l-FP-CIT and the response to levodopa therapy in VP subjects. | 9/20 had normal scan findings and poor response to levodopa, 6/20 had abnormal normal scan findings and no response to levodopa, 5/20 had abnormal scan findings and good response to levodopa. | VP subjects had a poor response to levodopa. Normal ^123^l-FP-CIT FP-CIT SPECT may predict a poor response to levodopa. |
| Ondo et al., (2002) (32).  United States of America. | Pilot study. | ***Lumbar puncture (1 puncture).*** | 40 VP subjects. | P&DSWML. | To determine factors that may predict improvement from CSF drainage in VP subjects. | LP showed a subjective improvement: significant improvement in 15/40 subjects (37.5%), mild improvement in 13/40 subjects (32.5%) and no improvement in 12/40 subjects (30%). Mean duration of response was 2.4±1.2 months. Deep subcortical WML improved in 1 subject after LP. | CSF drainage provoked subjective improvement in 28/40 VP subjects. Subjects responsive to LP had better response to levodopa. |
| Huang et al., (2002) (33).  United States of America. | Cross-sectional. | ***Levodopa (not reported).*** | 22 VP subjects. | Deep subcortical WML, LS in BG and pons. | To investigate if anticardiolipin antibodies are associated with VP. | 3/18 VP subjects demonstrated subjective levodopa responsiveness. | Levodopa treatment showed a poor subjective response in VP subjects. |
| Winikates et al., (1999) (16).  United States of America. | Cross-sectional. | ***Levodopa (not reported).*** | 346 subjects: 69 VP, 277 PD. | Involvement of multiple vascular territories, P&DSWML and LS in BG and brainstem. | To evaluate parkinsonian subjects for evidence of vascular disease and compare the clinical features in VP and PD. | 24.6% of the VP subjects treated with levodopa responded compared with 73.6% of PD subjects. | VP subjects were less likely to respond to levodopa than PD subjects. |
| 123l –labelled fluoropropyl-2b-carbomethoxy-3b-(4-iodophenyl)-tropane single photon emission computed tomography (123l-FP-CIT SPECT), basal ganglia (BG), cerebrospinal fluid (CSF), healthy subjects (HS), lacunar stroke (LS), meta-iodobenzylguanidine (MIBG ), Parkinson´s disease (PD), periventricular & deep subcortical white matter lesions (P&DSWML), repetitive transcranial magnetic stimulation (rTMS), timed 10 m walk (T10MW), Unified Parkinson´s Disease Rating Scale (UPDRS), vascular parkinsonism (VP), white matter lesions (WML). | | | | | | | |

**Table S2. VP studies with Winikates´ criteria diagnosis.**

| **Author & country** | **Type of study** | **Treatment (duration for VP)** | **Population** | **Purpose of the study** | **VP diagnosis** | **Results for VP and treatment** | **Conclusion for VP and treatment** |
| --- | --- | --- | --- | --- | --- | --- | --- |
| Rampello et al., (2005) (34).  Italy. | Cohort. | ***Levodopa (not reported).*** | 77 subjects: 45 VP, 32 PD. | To compare the clinical picture between PD and VP subjects. | 2/4 cardinal features of parkinsonism, vascular lesions on MRI and exclusion of other causes. | 17/45 (38%) of VP subjects had a good response to levodopa while 100% of PD patients did respond. | VP had a poor response to levodopa compared with PD subjects. |
| Demirkiran et al., (2001) (35).  Turkey. | Cross- sectional. | ***Levodopa (not reported).*** | 66 subjects: 16 VP, 50 PD. | To define the clinical features of suspected VP. | 2/4 cardinal features of parkinsonism, vascular lesions on MRI and exclusion of other causes. | 37% of VP patients had a moderate response to therapy. | Poor response to levodopa was an important distinguishing feature of VP. |
| Yamanouchi et al., (1997) (36).  Japan. | Clinicopathological. | ***Levodopa (not reported).*** | 76 subjects: 24 VP, 30 PD, 22 BD. | To investigate the clinical and neuropathologic characteristics of VP in autopsy cases. | 2/4 cardinal features of parkinsonism, evidence of cerebrovascular lesions, no depigmentation or Lewy bodies in substantia nigra, exclusion of other causes of parkinsonism. | 0/ 15 VP patients had a good response to treatment compared with 18/29 of PD patients. | VP was characterized with a negative response to levodopa. |
| Zijlman et al., (1996) (37).  Netherlands. | Cross- sectional. | ***Levodopa (not reported).*** | 34 subjects: 12 VP, 12 PD and 10 controls. | To investigate the differences on the gait pattern of VP subjects compared to PD subjects and controls. | At least 60 years old, hypokinesia and rigidity dominated by frontal gait disorder and exclusion of other causes of parkinsonism. | 1/12 showed a good response to levodopa while 11/12 showed absent or minimal response. | No response to levodopa. |
| Zijlmans et al., (1995) (38).  Netherlands. | Cross- sectional. | ***Levodopa (not reported).*** | 40 subjects: 15 VP, 15 PD and 10 hypertensive subjects. | To investigate if MRI can yield evidence of vascular lesions in suspected VP subjects and compare the scans with PD and hypertensive subjects. | At least 60 years old, hypokinesia and rigidity dominated by frontal gait disorder and exclusion of other causes of parkinsonism. | 1/15 showed a good response to levodopa while 14/15 showed absent or minimal response. | No response to levodopa. |
| Binswager disease (BD), computed tomography (CT), lacunar strokes (LS), magnetic resonance imaging (MRI), Parkinson´s disease (PD), vascular parkinsonism (VP). | | | | | | | |

**Table S3. VP studies without standardized diagnosis criteria.**

| **Table S4. Quality subanalysis VP event rate** | | | | | | | | | | | | | | | | | | |
| --- | --- | --- | --- | --- | --- | --- | --- | --- | --- | --- | --- | --- | --- | --- | --- | --- | --- | --- |
| **Subgroups** |  | **Effect size and 95% interval** | | | |  | **Test of null**  **(2- Tail)** | |  | **Heterogeneity** | | | |  | **Tau-squared** | | | |
|  |  | **Number**  **of studies** | **OR** | **Lower limit** | **Upper limit** |  | **z- value** | **P-value** |  | **Q-value** | **df (Q)** | **P-value** | **I^2^** |  | **Tau ^2^** | **Standard error** | **Variance** | **Tau** |
| **Fixed effect** |  |  |  |  |  |  |  |  |  |  |  |  |  |  |  |  |  |  |
|  |  |  |  |  |  |  |  |  |  |  |  |  |  |  |  |  |  |  |
| High quality |  | 4 | 0.345 | 0.285 | 0.410 |  | -4.505 | <0.001 |  | 11.161 | 3 | 0.011 | 73.12 |  | 0.243 | 0.295 | 0.087 | 0.493 |
| Low quality |  | 13 | 0.323 | 0.262 | 0.392 |  |  | <0.001 |  | 30.048 | 12 | 0.003 | 60.06 |  | 0.482 | 0.360 | 0.130 | 0.694 |
| Total within |  |  |  |  |  |  |  |  |  | 41.209 | 15 | <0.001 |  |  |  |  |  |  |
| Total between |  |  |  |  |  |  |  |  |  | 0.212 | 1 | 0.645 |  |  |  |  |  |  |
| Overall |  | 17 | 0.335 | 0.291 | 0.381 |  | -6.600 | <0.001 |  | 41.421 | 16 | <0.001 | 61.37 |  | 0.313 | 0.211 | 0.044 | 0.559 |
|  |  |  |  |  |  |  |  |  |  |  |  |  |  |  |  |  |  |  |
| **Random Effect** |  |  |  |  |  |  |  |  |  |  |  |  |  |  |  |  |  |  |
| High quality |  | 4 | 0.310 | 0.183 | 0.474 |  | -2.258 | 0.024 |  |  |  |  |  |  |  |  |  |  |
| Low quality |  | 13 |  |  |  |  | -3.527 | <0.001 |  |  |  |  |  |  |  |  |  |  |
| Total between |  |  |  |  |  |  |  |  |  | 0.024 | 1 | 0.877 |  |  |  |  |  |  |
| Overall |  | 17 | 0.301 | 0.224 | 0.390 |  | -4.184 | <0.001 |  |  |  |  |  |  |  |  |  |  |

| **Table S5. Continent subanalysis VP event rate** | | | | | | | | | | | | | | | | | | |
| --- | --- | --- | --- | --- | --- | --- | --- | --- | --- | --- | --- | --- | --- | --- | --- | --- | --- | --- |
| **Subgroups** |  | **Effect size and 95% interval** | | | |  | **Test of null**  **(2- Tail)** | |  | **Heterogeneity** | | | |  | **Tau-squared** | | | |
|  |  | **Number**  **of studies** | **OR** | **Lower limit** | **Upper limit** |  | **z- value** | **P-value** |  | **Q-value** | **df (Q)** | **P-value** | **I^2^** |  | **Tau ^2^** | **Standard error** | **Variance** | **Tau** |
| **Fixed effect** |  |  |  |  |  |  |  |  |  |  |  |  |  |  |  |  |  |  |
|  |  |  |  |  |  |  |  |  |  |  |  |  |  |  |  |  |  |  |
| America |  | 2 | 0.234 | 0.156 | 0.335 |  | -4.638 | <0.001 |  | 0.370 | 1 | 0.543 | 0.00 |  | 0.00 | 0.341 | 0.117 | 0.00 |
| Asia |  | 5 | 0.241 | 0.161 | 0.345 |  | -4.452 | <0.001 |  | 8.978 | 4 | 0.062 | 55.45 |  | 0.496 | 0.692 | 0.479 | 0.704 |
| Europe |  | 10 | 0.389 | 0.332 | 0.449 |  | -3.557 | <0.001 |  | 21.653 | 9 | 0.010 | 58.43 |  | 0.255 | 0.237 | 0.056 | 0.505 |
| Total within |  |  |  |  |  |  |  |  |  | 31.000 | 14 | 0.006 |  |  |  |  |  |  |
| Total between |  |  |  |  |  |  |  |  |  | 10.421 | 2 | 0.005 |  |  |  |  |  |  |
| Overall |  | 17 | 0.335 | 0.291 | 0.381 |  | -6.600 | <0.001 |  | 41.421 | 16 | <0.001 | 61.37 |  | 0.313 | 0.211 | 0.044 | 0.559 |
|  |  |  |  |  |  |  |  |  |  |  |  |  |  |  |  |  |  |  |
| **Random Effect** |  |  |  |  |  |  |  |  |  |  |  |  |  |  |  |  |  |  |
| America |  | 2 | 0.221 | 0.100 | 0.419 |  | -2.643 | 0.008 |  |  |  |  |  |  |  |  |  |  |
| Asia |  | 5 | 0.225 | 0.121 | 0.373 |  | -3.255 | 0.001 |  |  |  |  |  |  |  |  |  |  |
| Europe |  | 10 | 0.365 | 0.267 | 0.475 |  | -2.389 | 0.017 |  |  |  |  |  |  |  |  |  |  |
| Total between |  |  |  |  |  |  |  |  |  | 3.366 | 2 | 0.186 |  |  |  |  |  |  |
| Overall |  | 17 | 0.282 | 0.179 | 0.414 |  | -3.116 | 0.002 |  |  |  |  |  |  |  |  |  |  |

| **Table S6. Type of study subanalysis VP event rate** | | | | | | | | | | | | | | | | | | |
| --- | --- | --- | --- | --- | --- | --- | --- | --- | --- | --- | --- | --- | --- | --- | --- | --- | --- | --- |
| **Subgroups** |  | **Effect size and 95% interval** | | | |  | **Test of null**  **(2- Tail)** | |  | **Heterogeneity** | | | |  | **Tau-squared** | | | |
|  |  | **Number**  **of studies** | **OR** | **Lower limit** | **Upper limit** |  | **z- value** | **P-value** |  | **Q-value** | **df (Q)** | **P-value** | **I^2^** |  | **Tau ^2^** | **Standard error** | **Variance** | **Tau** |
| **Fixed effect** |  |  |  |  |  |  |  |  |  |  |  |  |  |  |  |  |  |  |
|  |  |  |  |  |  |  |  |  |  |  |  |  |  |  |  |  |  |  |
| Case control |  | 2 | 0.465 | 0.363 | 0.571 |  | -0.638 | 0.523 |  | 0.396 | 1 | 0.529 | 0.00 |  | 0.00 | 0.269 | 0.072 | 0.00 |
| Cohort |  | 2 | 0.339 | 0.261 | 0.428 |  | -3.459 | 0.001 |  | 0.488 | 1 | 0.487 | 0.00 |  | 0.00 | 0.110 | 0.012 | 0.00 |
| Cross sectional |  | 13 | 0.275 | 0.220 | 0.338 |  | -6.407 | <0.001 |  | 30.671 | 12 | 0.002 | 60.88 |  | 0.499 | 0.378 | 0.143 | 0.706 |
| Total within |  |  |  |  |  |  |  |  |  | 31.550 | 14 | 0.005 |  |  |  |  |  |  |
| Total between |  |  |  |  |  |  |  |  |  | 9.871 | 2 | 0.007 |  |  |  |  |  |  |
| Overall |  | 17 | 0.335 | 0.291 | 0.381 |  | -6.600 | <0.001 |  | 41.421 | 16 | <0.001 | 61.37 |  | 0.313 | 0.211 | 0.044 | 0.559 |
|  |  |  |  |  |  |  |  |  |  |  |  |  |  |  |  |  |  |  |
| **Random Effect** |  |  |  |  |  |  |  |  |  |  |  |  |  |  |  |  |  |  |
| Case control |  | 2 | 0.444 | 0.236 | 0.674 |  | -0.463 | 0.643 |  |  |  |  |  |  |  |  |  |  |
| Cohort |  | 2 | 0.345 | 0.181 | 0.556 |  | -1.448 | 0.148 |  |  |  |  |  |  |  |  |  |  |
| Cross sectional |  | 13 | 0.263 | 0.183 | 0.362 |  | -4.335 | <0.001 |  |  |  |  |  |  |  |  |  |  |
| Total between |  |  |  |  |  |  |  |  |  | 2.459 | 2 | 0.293 |  |  |  |  |  |  |
| Overall |  | 17 | 0.324 | 0.215 | 0.455 |  | -2.596 | 0.009 |  |  |  |  |  |  |  |  |  |  |

| **Table S7. VP diagnosis subanalysis VP event rate** | | | | | | | | | | | | | | | | | | |
| --- | --- | --- | --- | --- | --- | --- | --- | --- | --- | --- | --- | --- | --- | --- | --- | --- | --- | --- |
| **Subgroups** |  | **Effect size and 95% interval** | | | |  | **Test of null**  **(2- Tail)** | |  | **Heterogeneity** | | | |  | **Tau-squared** | | | |
|  |  | **Number**  **of studies** | **OR** | **Lower limit** | **Upper limit** |  | **z- value** | **P-value** |  | **Q-value** | **df (Q)** | **P-value** | **I^2^** |  | **Tau ^2^** | **Standard error** | **Variance** | **Tau** |
| **Fixed effect** |  |  |  |  |  |  |  |  |  |  |  |  |  |  |  |  |  |  |
|  |  |  |  |  |  |  |  |  |  |  |  |  |  |  |  |  |  |  |
| Other |  | 5 | 0.308 | 0.216 | 0.418 |  | -3.312 | 0.001 |  | 10.128 | 4 | 0.038 | 60.50 |  | 0.682 | 0.918 | 0.843 | 0.826 |
| Winikates |  | 5 | 0.249 | 0.180 | 0.335 |  | -5.201 | <0.001 |  | 5.984 | 4 | 0.200 | 33.15 |  | 0.146 | 0.319 | 0.102 | 0.382 |
| Zijlmans |  | 7 | 0.383 | 0.322 | 0.449 |  | -3.468 | 0.001 |  | 18.817 | 6 | 0.004 | 68.11 |  | 0.318 | 0.306 | 0.094 | 0.563 |
| Total within |  |  |  |  |  |  |  |  |  | 34.928 | 14 | 0.002 |  |  |  |  |  |  |
| Total between |  |  |  |  |  |  |  |  |  | 6.493 | 2 | 0.039 |  |  |  |  |  |  |
| Overall |  | 17 | 0.335 | 0.291 | 0.381 |  | -6.600 | <0.001 |  | 41.421 | 16 | <0.001 | 61.38 |  | 0.313 | 0.211 | 0.044 | 0.559 |
|  |  |  |  |  |  |  |  |  |  |  |  |  |  |  |  |  |  |  |
| **Random Effect** |  |  |  |  |  |  |  |  |  |  |  |  |  |  |  |  |  |  |
| Other |  | 5 | 0.231 | 0.118 | 0.404 |  | -2.904 | 0.004 |  |  |  |  |  |  |  |  |  |  |
| Winikates |  | 5 | 0.240 | 0.132 | 0.394 |  | -3.123 | 0.002 |  |  |  |  |  |  |  |  |  |  |
| Zijlmans |  | 7 | 0.379 | 0.262 | 0.513 |  | -1.776 | 0.076 |  |  |  |  |  |  |  |  |  |  |
| Total between |  |  |  |  |  |  |  |  |  | 3.065 | 2 | 0.216 |  |  |  |  |  |  |
| Overall |  | 17 | 0.290 | 0.188 | 0.417 |  | -3.121 | 0.002 |  |  |  |  |  |  |  |  |  |  |

| Table S8. Sensitivity analysis VP event rate | | | | | | | | | | |
| --- | --- | --- | --- | --- | --- | --- | --- | --- | --- | --- |
| Study removed |  | **Event Rate after study removed** |  | **Lower limit** |  | **Upper limit** |  | **Z-Value** |  | **p-Value** |
| Lee et al. 2015 |  | 0.312 |  | 0.234 |  | 0.402 |  | -3.928 |  | <0.001 |
| Navarro Otano et al. 2014 |  | 0.313 |  | 0.237 |  | 0.400 |  | -4.045 |  | <0.001 |
| Jang et al. 2014 |  | 0.294 |  | 0.219 |  | 0.381 |  | -4.372 |  | <0.001 |
| Benitez Rivero et al. 2013 |  | 0.288 |  | 0.216 |  | 0.373 |  | -4.623 |  | <0.001 |
| Antonini et al. 2012 |  | 0.297 |  | 0.217 |  | 0.393 |  | -3.960 |  | <0.001 |
| Zijlmans et al. 2007 |  | 0.297 |  | 0.222 |  | 0.386 |  | -4.248 |  | <0.001 |
| Zijlmans et al. 2004 |  | 0.287 |  | 0.220 |  | 0.364 |  | -5.061 |  | <0.001 |
| Kim et al. 2006 |  | 0.315 |  | 0.242 |  | 0.398 |  | -4.182 |  | <0.001 |
| Katzenschlanger et al. 2004 |  | 0.295 |  | 0.220 |  | 0.382 |  | -4.354 |  | <0.001 |
| Lorberboym et al. 2004 |  | 0.306 |  | 0.229 |  | 0.396 |  | -4.044 |  | <0.001 |
| Huang et al. 2002 |  | 0.312 |  | 0.235 |  | 0.400 |  | -4.019 |  | <0.001 |
| Winikates et al. 1999 |  | 0.308 |  | 0.229 |  | 0.400 |  | -3.906 |  | <0.001 |
| Rampello et al. 2005 |  | 0.294 |  | 0.216 |  | 0.386 |  | -4.156 |  | <0.001 |
| Demirkiran et al. 2001 |  | 0.297 |  | 0.221 |  | 0.387 |  | -4.225 |  | <0.001 |
| Yamanouchi et al. 1997 |  | 0.314 |  | 0.241 |  | 0.398 |  | -4.175 |  | <0.001 |
| Zijlmans et al. 1996 |  | 0.313 |  | 0.239 |  | 0.399 |  | -4.102 |  | <0.001 |
| Zijlmans et al. 1995 |  | 0.316 |  | 0.241 |  | 0.400 |  | -4.098 |  | <0.001 |
| Original analysis |  | 0.304 |  | 0.230 |  | 0.388 |  | -4.327 |  | <0.001 |

| **Table S9. Continent subanalysis PD *vs* VP** | | | | | | | | | | | | | | | | | | |
| --- | --- | --- | --- | --- | --- | --- | --- | --- | --- | --- | --- | --- | --- | --- | --- | --- | --- | --- |
| **Subgroups** |  | **Effect size and 95% interval** | | | |  | **Test of null**  **(2- Tail)** | |  | **Heterogeneity** | | | |  | **Tau-squared** | | | |
|  |  | **Number**  **of studies** | **OR** | **Lower limit** | **Upper limit** |  | **z- value** | **P-value** |  | **Q-value** | **df (Q)** | **P-value** | **I^2^** |  | **Tau ^2^** | **Standard error** | **Variance** | **Tau** |
| **Fixed effect** |  |  |  |  |  |  |  |  |  |  |  |  |  |  |  |  |  |  |
|  |  |  |  |  |  |  |  |  |  |  |  |  |  |  |  |  |  |  |
| America |  | 1 | 0.053 | 0.027 | 0.102 |  | -8.735 | <0.001 |  | 0.000 | 0 | 1.000 | 0.00 |  | 0.000 | 0.000 | 0.000 | 0.000 |
| Asia |  | 3 | 0.009 | 0.001 | 0.058 |  | -4.977 | <0.001 |  | 2.980 | 2 | 0.225 | 32.88 |  | 1.347 | 4.107 | 16.866 | 1.161 |
| Europe |  | 5 | 0.157 | 0.086 | 0.285 |  | -6.080 | <0.001 |  | 25.988 | 4 | <0.001 | 84.61 |  | 6.854 | 6.833 | 46.683 | 2.618 |
| Total within |  |  |  |  |  |  |  |  |  | 28.967 | 6 | <0.001 |  |  |  |  |  |  |
| Total between |  |  |  |  |  |  |  |  |  | 11.575 | 2 | 0.003 |  |  |  |  |  |  |
| Overall |  | 9 | 0.084 | 0.055 | 0.130 |  | -11.245 | <0.001 |  | 40.543 | 8 | <0.001 | 80.27 |  | 2.503 | 2.646 | 7.001 | 1.582 |
|  |  |  |  |  |  |  |  |  |  |  |  |  |  |  |  |  |  |  |
| **Random Effect** |  |  |  |  |  |  |  |  |  |  |  |  |  |  |  |  |  |  |
| America |  | 1 | 0.053 | 0.000 | 6.303 |  | -1.205 | 0.228 |  |  |  |  |  |  |  |  |  |  |
| Asia |  | 3 | 0.007 | 0.000 | 0.192 |  | -2.933 | 0.003 |  |  |  |  |  |  |  |  |  |  |
| Europe |  | 5 | 0.015 | 0.001 | 0.161 |  | -3.445 | 0.001 |  |  |  |  |  |  |  |  |  |  |
| Total between |  |  |  |  |  |  |  |  |  | 0.472 | 2 | 0.790 |  |  |  |  |  |  |
| Overall |  | 9 | 0.014 | 0.002 | 0.085 |  | -4.632 | <0.001 |  |  |  |  |  |  |  |  |  |  |

| **Table S10. Type of study subanalysis PD *vs* VP** | | | | | | | | | | | | | | | | | | |
| --- | --- | --- | --- | --- | --- | --- | --- | --- | --- | --- | --- | --- | --- | --- | --- | --- | --- | --- |
| **Subgroups** |  | **Effect size and 95% interval** | | | |  | **Test of null**  **(2- Tail)** | |  | **Heterogeneity** | | | |  | **Tau-squared** | | | |
|  |  | **Number**  **of studies** | **OR** | **Lower limit** | **Upper limit** |  | **z- value** | **P-value** |  | **Q-value** | **df (Q)** | **P-value** | **I^2^** |  | **Tau ^2^** | **Standard error** | **Variance** | **Tau** |
| **Fixed effect** |  |  |  |  |  |  |  |  |  |  |  |  |  |  |  |  |  |  |
|  |  |  |  |  |  |  |  |  |  |  |  |  |  |  |  |  |  |  |
| Case control |  | 1 | 0.002 | 0.000 | 0.032 |  | -4.364 | <0.001 |  | 0 | 0 | 1.000 | 0.00 |  | 0.000 | 0.000 | 0.000 | 0.000 |
| Cohort |  | 2 | 0.261 | 0.138 | 0.494 |  | -4.124 | <0.001 |  | 5.462 | 1 | 0.019 | 81.69 |  | 4.985 | 8.630 | 74.483 | 2.223 |
| Cross sectional |  | 6 | 0.037 | 0.021 | 0.068 |  | -10.797 | <0.001 |  | 8.917 | 5 | 0.112 | 43.93 |  | 1.123 | 1.737 | 3.016 | 1.060 |
| Total within |  |  |  |  |  |  |  |  |  | 14.379 | 6 | 0.026 |  |  |  |  |  |  |
| Total between |  |  |  |  |  |  |  |  |  | 26.164 | 2 | <0.001 |  |  |  |  |  |  |
| Overall |  | 9 | 0.084 | 0.055 | 0.130 |  | -11.245 | <0.001 |  | 40.543 | 8 | <0.001 | 80.27 |  | 2.503 | 2.646 | 7.001 | 1.582 |
|  |  |  |  |  |  |  |  |  |  |  |  |  |  |  |  |  |  |  |
| **Random Effect** |  |  |  |  |  |  |  |  |  |  |  |  |  |  |  |  |  |  |
| Case control |  | 1 | 0.002 | 0.000 | 0.095 |  | -3.142 | 0.002 |  |  |  |  |  |  |  |  |  |  |
| Cohort |  | 2 | 0.097 | 0.010 | 0.942 |  | -2.012 | 0.044 |  |  |  |  |  |  |  |  |  |  |
| Cross sectional |  | 6 | 0.013 | 0.003 | 0.062 |  | -5.476 | <0.001 |  |  |  |  |  |  |  |  |  |  |
| Total between |  |  |  |  |  |  |  |  |  | 3.507 | 2 | 0.173 |  |  |  |  |  |  |
| Overall |  | 9 | 0.018 | 0.018 | 0.146 |  | -3.783 | <0.001 |  |  |  |  |  |  |  |  |  |  |

| **Table S11. Quality subanalysis PD *vs* VP** | | | | | | | | | | | | | | | | | | |
| --- | --- | --- | --- | --- | --- | --- | --- | --- | --- | --- | --- | --- | --- | --- | --- | --- | --- | --- |
| **Subgroups** |  | **Effect size and 95% interval** | | | |  | **Test of null**  **(2- Tail)** | |  | **Heterogeneity** | | | |  | **Tau-squared** | | | |
|  |  | **Number**  **of studies** | **OR** | **Lower limit** | **Upper limit** |  | **z- value** | **P-value** |  | **Q-value** | **df (Q)** | **P-value** | **I^2^** |  | **Tau ^2^** | **Standard error** | **Variance** | **Tau** |
| **Fixed effect** |  |  |  |  |  |  |  |  |  |  |  |  |  |  |  |  |  |  |
|  |  |  |  |  |  |  |  |  |  |  |  |  |  |  |  |  |  |  |
| High quality |  | 4 | 0.109 | 0.069 | 0.171 |  | -9.593 | <0.001 |  | 25.546 | 3 | <0.001 | 88.26 |  | 2.217 | 2.921 | 8.531 | 1.489 |
| Low quality |  | 5 | 0.008 | 0.002 | 0.033 |  | -6.811 | <0.001 |  | 3.039 | 4 | 0.551 | 0.00 |  | 0.000 | 1.754 | 3.075 | 0.000 |
| Total within |  |  |  |  |  |  |  |  |  | 28.585 | 7 | <0.001 |  |  |  |  |  |  |
| Total between |  |  |  |  |  |  |  |  |  | 11.958 | 1 | 0.001 |  |  |  |  |  |  |
| Overall |  | 9 | 0.084 | 0.055 | 0.130 |  | -11.245 | <0.001 |  | 40.543 | 8 | <0.001 | 80.27 |  | 2.503 | 2.646 | 7.001 | 1.582 |
|  |  |  |  |  |  |  |  |  |  |  |  |  |  |  |  |  |  |  |
| **Random Effect** |  |  |  |  |  |  |  |  |  |  |  |  |  |  |  |  |  |  |
| High quality |  | 4 | 0.040 | 0.008 | 0.197 |  | -3.961 | <0.001 |  |  |  |  |  |  |  |  |  |  |
| Low quality |  | 5 | 0.008 | 0.001 | 0.048 |  | -5.218 | <0.001 |  |  |  |  |  |  |  |  |  |  |
| Total between |  |  |  |  |  |  |  |  |  | 1.761 | 1 | 0.184 |  |  |  |  |  |  |
| Overall |  | 9 | 0.019 | 0.004 | 0.093 |  | -4.862 | <0.001 |  |  |  |  |  |  |  |  |  |  |

| **Table S12. VP diagnosis subanalysis PD *vs* VP** | | | | | | | | | | | | | | | | | | |
| --- | --- | --- | --- | --- | --- | --- | --- | --- | --- | --- | --- | --- | --- | --- | --- | --- | --- | --- |
| **Subgroups** |  | **Effect size and 95% interval** | | | |  | **Test of null**  **(2- Tail)** | |  | **Heterogeneity** | | | |  | **Tau-squared** | | | |
|  |  | **Number**  **of studies** | **OR** | **Lower limit** | **Upper limit** |  | **z- value** | **P-value** |  | **Q-value** | **df (Q)** | **P-value** | **I^2^** |  | **Tau ^2^** | **Standard error** | **Variance** | **Tau** |
| **Fixed effect** |  |  |  |  |  |  |  |  |  |  |  |  |  |  |  |  |  |  |
|  |  |  |  |  |  |  |  |  |  |  |  |  |  |  |  |  |  |  |
| Other |  | 3 | 0.011 | 0.002 | 0.056 |  | -5.318 | <0.001 |  | 0.323 | 2 | 0.851 | 0.00 |  | 0.000 | 2.198 | 4.833 | 0.000 |
| Winikates |  | 2 | 0.046 | 0.024 | 0.089 |  | -9.248 | <0.001 |  | 5.569 | 1 | 0.018 | 82.04 |  | 9.589 | 16.529 | 273.200 | 3.097 |
| Zijlmans |  | 4 | 0.189 | 0.103 | 0.349 |  | -5.336 | <0.001 |  | 18.821 | 3 | <0.001 | 84.06 |  | 6.620 | 7.317 | 53.540 | 2.573 |
| Total within |  |  |  |  |  |  |  |  |  | 24.713 | 6 | <0.001 |  |  |  |  |  |  |
| Total between |  |  |  |  |  |  |  |  |  | 15.830 | 2 | <0.001 |  |  |  |  |  |  |
| Overall |  | 9 | 0.084 | 0.055 | 0.130 |  | -11.245 | <0.001 |  | 40.543 | 8 | <0.001 | 80.27 |  | 2.503 | 2.646 | 7.001 | 1.582 |
|  |  |  |  |  |  |  |  |  |  |  |  |  |  |  |  |  |  |  |
| **Random Effect** |  |  |  |  |  |  |  |  |  |  |  |  |  |  |  |  |  |  |
| Other |  | 3 | 0.011 | 0.002 | 0.056 |  | -5.318 | <0.001 |  |  |  |  |  |  |  |  |  |  |
| Winikates |  | 2 | 0.007 | 0.000 | 0.758 |  | .2.076 | 0.038 |  |  |  |  |  |  |  |  |  |  |
| Zijlmans |  | 4 | 0.021 | 0.001 | 0.356 |  | -2.678 | 0.007 |  |  |  |  |  |  |  |  |  |  |
| Total between |  |  |  |  |  |  |  |  |  | 0.231 | 2 | 0.891 |  |  |  |  |  |  |
| Overall |  | 9 | 0.102 | 0.003 | 0.048 |  | -6.288 | <0.001 |  |  |  |  |  |  |  |  |  |  |

| Table S13. Sensitivity analysis PD *vs* VP | | | | | | | | | | |
| --- | --- | --- | --- | --- | --- | --- | --- | --- | --- | --- |
| Study removed |  | **Event Rate after study removed** |  | **Lower limit** |  | **Upper limit** |  | **Z-Value** |  | **p-Value** |
| Navarro Otano et al. 2014 |  | 0.019 |  | 0.005 |  | 0.078 |  | -5.560 |  | <0.001 |
| Jang et al. 2014 |  | 0.017 |  | 0.004 |  | 0.069 |  | -5.639 |  | <0.001 |
| Benitez Rivero et al. 2013 |  | 0.024 |  | 0.006 |  | 0.090 |  | -5.515 |  | <0.001 |
| Antonini et al. 2012 |  | 0.011 |  | 0.003 |  | 0.038 |  | -7.190 |  | <0.001 |
| Kim et al. 2006 |  | 0.024 |  | 0.007 |  | 0.089 |  | -5.608 |  | <0.001 |
| Winikates et al. 1999 |  | 0.011 |  | 0.002 |  | 0.080 |  | -4.464 |  | <0.001 |
| Rampello et al. 2005 |  | 0.019 |  | 0.005 |  | 0.076 |  | -5.552 |  | <0.001 |
| Demirkiran et al. 2001 |  | 0.020 |  | 0.005 |  | 0.079 |  | -5.540 |  | <0.001 |
| Yamanouchi et al. 1997 |  | 0.017 |  | 0.004 |  | 0.071 |  | -5.616 |  | <0.001 |
| Original analysis |  | 0.018 |  | 0.005 |  | 0.066 |  | -5.965 |  | <0.001 |

| Table S14. Sensitivity analysis PD *vs* VP | | | | | | | | | | |
| --- | --- | --- | --- | --- | --- | --- | --- | --- | --- | --- |
| Study removed |  | **Event Rate after study removed** |  | **Lower limit** |  | **Upper limit** |  | **Z-Value** |  | **p-Value** |
| Lee et al. 2015 |  | 15.524 |  | 4.558 |  | 52.870 |  | 4.386 |  | <0.001 |
| Antonini et al. 2012 |  | 16.150 |  | 3.665 |  | 71.166 |  | 3.676 |  | <0.001 |
| Zijlmans et al. 2004 |  | 14.361 |  | 4.464 |  | 46.198 |  | 4.469 |  | <0.001 |
| Lorberboym et al. 2004 |  | 15.023 |  | 4.794 |  | 47.076 |  | 4.650 |  | <0.001 |
| Original analysis |  | 15.148 |  | 5.195 |  | 44.169 |  | 4.978 |  | <0.001 |
